# Chiral phosphines in nucleophilic organocatalysis

**DOI:** 10.3762/bjoc.10.218

**Published:** 2014-09-04

**Authors:** Yumei Xiao, Zhanhu Sun, Hongchao Guo, Ohyun Kwon

**Affiliations:** 1 Department of Applied Chemistry, China Agricultural University, Beijing 100193, P. R. China; 2 Department of Chemistry and Biochemistry, University of California, Los Angeles, CA 90095, USA

**Keywords:** asymmetric catalysis, chiral phosphine, nucleophilic, organocatalysis, organophosphorus, synthesis

## Abstract

This review discusses the tertiary phosphines possessing various chiral skeletons that have been used in asymmetric nucleophilic organocatalytic reactions, including annulations of allenes, alkynes, and Morita–Baylis–Hillman (MBH) acetates, carbonates, and ketenes with activated alkenes and imines, allylic substitutions of MBH acetates and carbonates, Michael additions, γ-umpolung additions, and acylations of alcohols.

## Introduction

During the past two decades, tertiary phosphine catalysts have been applied extensively in a wide range of carbon–carbon and carbon–heteroatom bond-forming transformations [1–18]. Many phosphine-catalyzed reactions have been developed for the syntheses of various biologically important acyclic and cyclic molecules. Asymmetric variants of these reactions have evolved relatively slowly. Indeed, very little research on chiral tertiary phosphine-catalyzed asymmetric reactions occurred prior to the year 2000 [19–20]. Over the last decade, however, and especially since 2005, considerable progress has been made in asymmetric phosphine catalysis. As a result, phosphine-catalyzed asymmetric reactions are now powerful and versatile tools for the construction of C–C, C–N, C–O, and C–S bonds and for the syntheses of functionalized carbocycles and heterocycles [11,13–14]. In offering a general account of this field, herein we summarize the major developments in nucleophilic chiral phosphine-catalyzed asymmetric reactions, including annulations of allenes, ketenes, alkynes, and Morita–Baylis–Hillman (MBH) carbonates with activated alkenes and imines, allylic substitution of MBH acetates and carbonates, Michael additions, γ-umpolung additions, and acylations of alcohols. Our discussion is organized according to the structural features of the chiral phosphines, the reaction types, and the nature of the substrate. Because chiral phosphine-promoted Rauhut–Currier (RC) reactions [9–10] and MBH/aza-MBH reactions [21–26] have been summarized splendidly in several reviews, we do not cover these transformations, except for selected examples related to other reactions.

## Review

### 1 Chiral phosphine catalysts

Nucleophilic phosphine catalysis often involves the formation of Lewis adducts, namely phosphonium (di)enolate zwitterions, as reaction intermediates [1,3,6,17]. These intermediates are formed through nucleophilic attack of the phosphine catalysts at electron-poor nuclei (normally carbon atoms) and then proceed through several steps to form new chemical bonds. Generally, the efficiency of nucleophilic phosphine catalysis often depends on the nature of the tertiary phosphine. Although many reactions require more nucleophilic trialkylphosphines as catalysts, only a few chiral trialkylphosphines are available. The synthesis of novel trialkylphosphines can be quite difficult, thereby limiting the scope of their chiral variants. Moreover, because of inherent air-sensitivity, the storage of trialkylphosphines can be problematic. On the other hand, thousands of arylphosphines have been used as chiral ligands for metal-catalyzed asymmetric reactions [27–30]. Most of these phosphines are acyclic, usually possess low nucleophilic activity, and generally display poor enantioselectivities for phosphine organocatalysis. For example, 2,2´-bis(diphenylphosphino)-1,1´-binaphthyl (BINAP) is an excellent chiral diphosphine ligand for metal-catalyzed asymmetric reactions, but it displays extremely poor reactivity and enantioselectivity in many nucleophilic phosphine-catalyzed reactions. For these reasons, effective chiral catalysts for nucleophilic phosphine catalysis are scarce, seriously limiting the development of asymmetric variants. At present, the synthesis of new chiral phosphines designed specifically for nucleophilic organocatalysis remains a significant challenge.

In the early exploration stage of asymmetric nucleophilic phosphine organocatalysis, chiral phosphines that had originally been designed as ligands for metal-catalyzed reactions were selected and examined for their reactivity. Although several cyclic phosphines were found to have excellent catalytic activities and enantioselectivities, only a few acyclic phosphines were effective. In this context, inspired by polyfunctional chiral small-molecule catalysts, particularly amino acid and thiourea-based systems [31–32], multifunctional chiral phosphines were constructed by installing a nucleophilic phosphine and a hydrogen bonding moiety on a molecular chiral backbone. These units served as active functional groups to synergistically activate the substrates in an assembled chiral environment, providing excellent catalytic activities and enantioselectivities that could not be accomplished using conventional chiral phosphines lacking hydrogen bonding moieties. Such multifunctional phosphines are readily accessible from simple chiral starting materials through a molecular building block approach, allowing combinatorial syntheses of new multifunctional chiral phosphines with diversity and, consequently, improving the probability of discovering an excellent catalyst.

**Figure 1:**
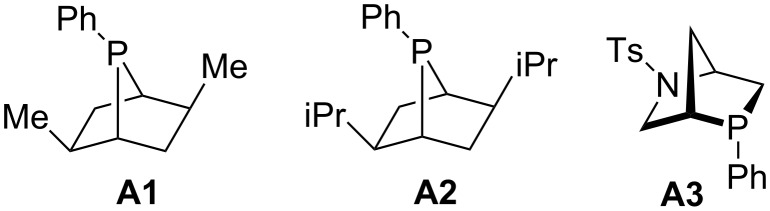
Cyclic chiral phosphines based on bridged-ring skeletons.

**Figure 2:**
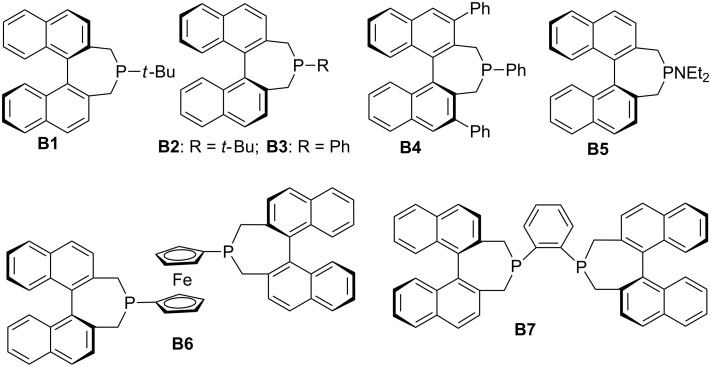
Cyclic chiral phosphines based on binaphthyl skeletons.

**Figure 3:**
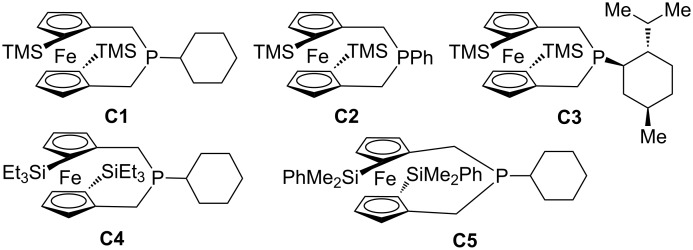
Cyclic chiral phosphines based on ferrocene skeletons.

**Figure 4:**
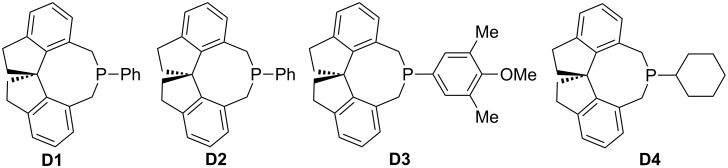
Cyclic chiral phosphines based on spirocyclic skeletons.

**Figure 5:**
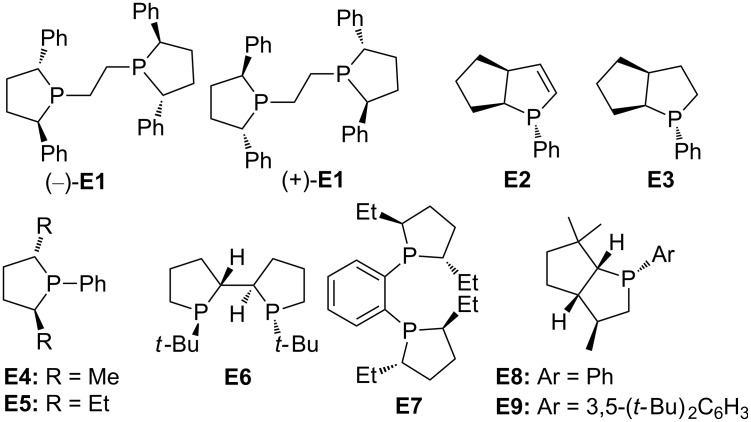
Cyclic chiral phosphines based on phospholane ring skeletons.

**Figure 6:**
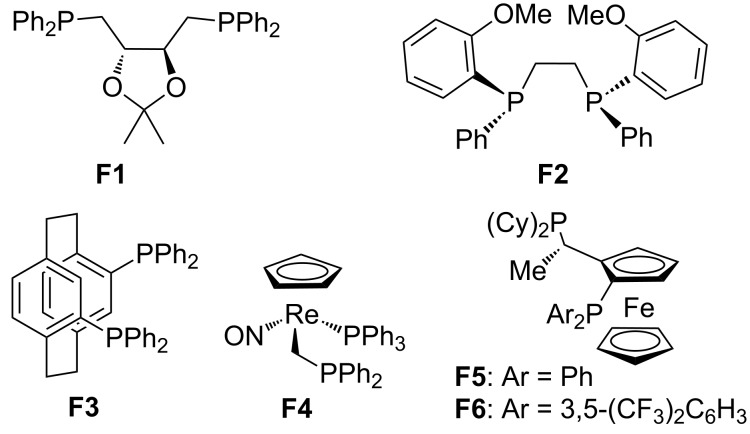
Acyclic chiral phosphines.

**Figure 7:**
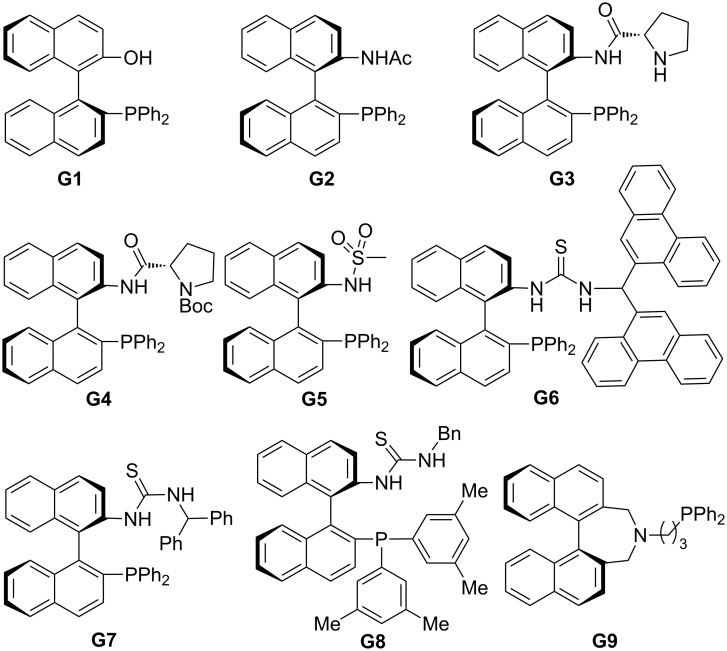
Multifunctional chiral phosphines based on binaphthyl skeletons.

**Figure 8:**
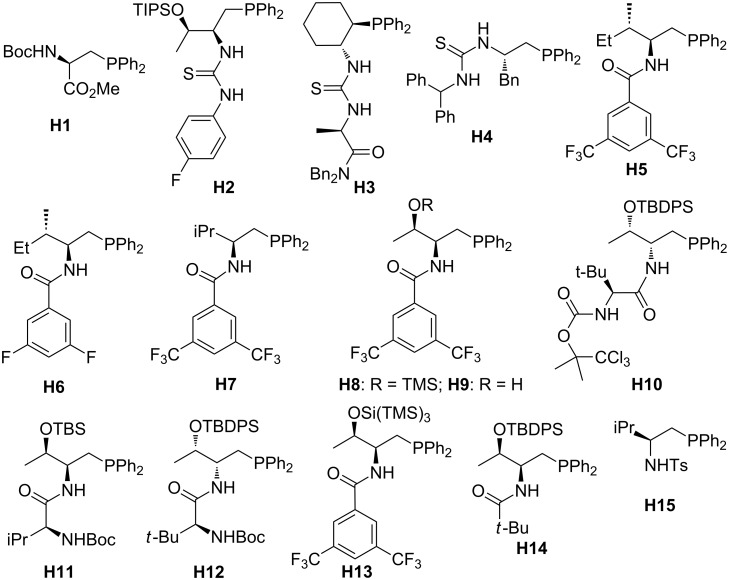
Multifunctional chiral phosphines based on amino acid skeletons.

In this review, we divide chiral phosphines into three classes: cyclic phosphines (Figures 1–5), acyclic phosphines (Figure 6), and multifunctional acyclic phosphines (Figures 7 and 8). Generally, the cyclic phosphines have been constructed based on bridged-ring (Figure 1), binaphthyl (Figure 2), ferrocene (Figure 3), spirocyclic (Figure 4), and five-membered phospholane ring (Figure 5) skeletons. Multifunctional chiral phosphines have generally been constructed based on binaphthyl skeletons (Figure 7) and amino acids (Figure 8). These structurally different chiral phosphines can be selected to catalyze appropriate asymmetric nucleophilic reactions. The electronic and steric properties of these phosphines can be tailored elaborately through modification of functional groups and the introduction of chiral elements, thereby providing suitable chirality, nucleophilicity, basicity, functionality, and rigidity.

### 2 Enantioselective reactions catalyzed by chiral phosphines

Using the chiral phosphines described above as catalysts, a number of highly enantioselective reactions have been achieved, offering a wide variety of methods for enantioselective syntheses of structurally diverse acyclic, carbocyclic, and heterocyclic compounds.

#### 2.1 [3 + 2] Annulation of allenes with activated alkenes

Nucleophilic phosphine-catalyzed annulations have been established as very useful tools for the syntheses of carbo- and heterocycles from simple starting materials [1–17]. Although a diverse set of annulations, including [2 + 1], [2 + 2], [4 + 1], [3 + 2], [2 + 2 + 1], [2 + 2 + 2], [3 + 2 + 3], [3 + 3], [4 + 2], [4 + 3], [6 + 3], [8 + 2], and [8 + 3] annulations, has been developed, asymmetric variants exist for only a few of them. In particular, chiral phosphine-catalyzed [3 + 2] annulations of allenes, alkynes, and MBH adducts with electron-deficient olefins and imines, resulting in cyclopentenes and pyrrolidines, have been the most studied, with many successful reported examples.

**Scheme 1:**
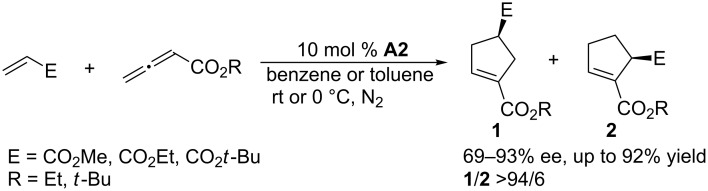
Asymmetric [3 + 2] annulations of allenoates with electron-deficient olefins, catalyzed by the chiral bicyclic phosphine **A2**.

**2.1.1 [3 + 2] Annulations using cyclic phosphines as chiral catalysts:** The first appearance in the literature of a chiral phosphine-catalyzed annulation was reported by Zhang and co-workers in 1997 [33]. Based on Lu’s work on phosphine-catalyzed annulation [34], Zhang et al. employed (Scheme 1) a chiral bicyclic phosphine **A2** to achieve asymmetric [3 + 2] annulations between several allenoates and electron-deficient olefins in benzene at room temperature with excellent regioselectivities (**1**:**2** >94/6) and enantioselectivities (**1**, 69–93% ee). This catalyst features a rigid bridged [2.2.1] bicyclic structure. The excellent regioselectivities and enantioselectivities resulted from the existence of the two isopropyl substituents in the chiral phosphine **A2**, which effectively controlled the approach of the acrylate toward the plausible phosphine/allenoate zwitterionic intermediate. Although the yields were not always very high, this inspiring study provided invaluable insight into asymmetric annulations catalyzed by chiral phosphines.

**Scheme 2:**
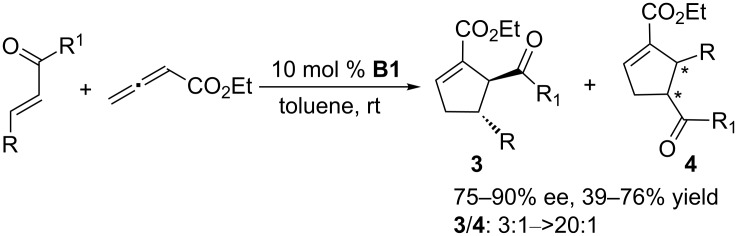
Asymmetric [3 + 2] annulations of allenoate and enones, catalyzed by the chiral binaphthyl-based phosphepine **B1**.

Until 2006, almost one decade later, no reports appeared of novel phosphine-catalyzed asymmetric [3 + 2] annulations. Then, using a chiral phosphepine **B1** based on a binaphthyl skeleton, Fu and co-workers developed the first asymmetric [3 + 2] annulation of ethyl allenoate with various α,β-unsaturated enones to provide functionalized cyclopentenes (Scheme 2) [35]. The key structural feature of the chiral catalyst **B1** is its rigid binaphthyl skeleton. This approach allowed the preparation of a wide array of cyclopentenes with two adjacent chiral centers, with good enantiomeric excesses and satisfactory to good regioselectivities. These transformations were slightly influenced by electronic effects. Notably, Fu et al. successfully applied this approach to construct spirocyclic compounds containing two neighboring quaternary and tertiary stereocenters in modest to excellent yields (up to 97%) and high enantioselectivities (up to 95% ee).

**Scheme 3:**
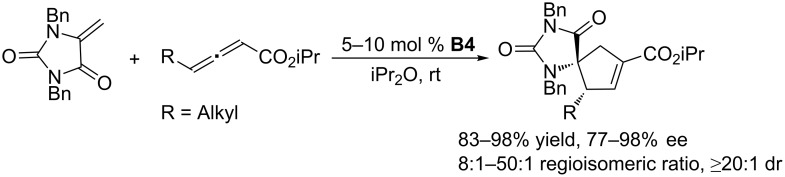
Asymmetric [3 + 2] annulations of *N*-substituted olefins and allenoates, catalyzed by the chiral binaphthyl-based phosphepine **B4**.

Subsequently, the Fu group applied this approach to the [3 + 2] annulation of allenes with 1,1-disubstituted olefins to synthesize highly functionalized cyclopentenes that bear an array of heteroatom-substituted quaternary stereocenters [36]. From a screening of catalysts, they carefully examined the effect of substitution of the binaphthyl framework of chiral phosphines, identifying the 3,3´-diphenyl-substituted phosphepine **B4** as the optimal catalyst. Based on the X-ray crystal structure of the catalyst, they proposed that the chiral microenvironment of the binaphthyl-based phosphepine was amplified by its 3,3´-diphenyl substituents. In the presence of the chiral phosphepine **B4**, the reactions of allenes with electron-deficient 1,1-disubstituted olefins proceeded smoothly to give functionalized cyclopentenes in satisfactory yields with up to 98% ee (Scheme 3). That study extended the substrate scope of known asymmetric phosphine-catalyzed [3 + 2] annulation reactions to diverse heteroatom-substituted olefins and allenamides. Nitrogen-, phosphorus-, oxygen-, and sulfur-substituted olefins and allenamides were compatible with these **B4**-catalyzed reactions. Fu’s results provided useful hints for further expansion of the substrate scope.

**Scheme 4:**
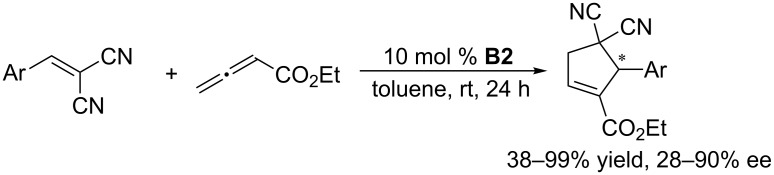
Asymmetric [3 + 2] annulations of 2-aryl-1,1-dicyanoethylenes with ethyl allenoate, catalyzed by the chiral binaphthyl-based phosphepine **B2**.

**Scheme 5:**
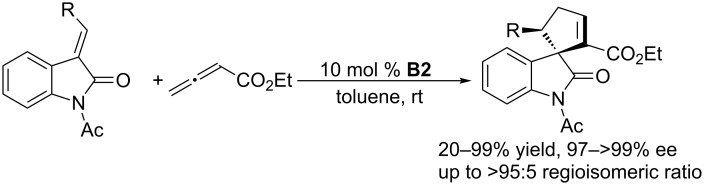
Asymmetric [3 + 2] annulations of 3-alkylideneindolin-2-ones with ethyl allenoate, catalyzed by the chiral binaphthyl-based phosphepine **B2**.

**Scheme 6:**
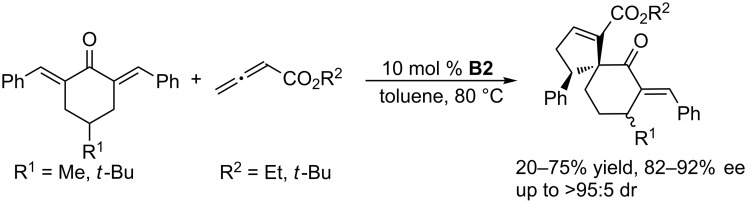
Asymmetric [3 + 2] annulations of 2,6-diarylidenecyclohexanones with allenoates, catalyzed by the chiral binaphthyl-based phosphepine **B2**.

**Scheme 7:**
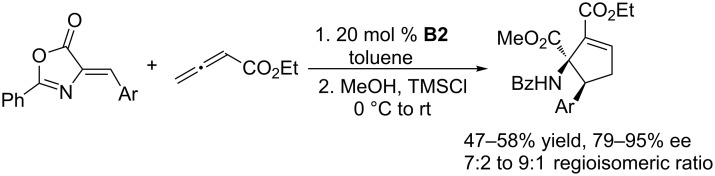
Asymmetric [3 + 2] annulations of allenoate with alkylidene azlactones, catalyzed by the chiral binaphthyl-based phosphepine **B2**, and subsequent alcoholysis in methanol.

Using **B2** as the chiral catalyst, Marinetti and co-workers also developed several asymmetric [3 + 2] annulations of allenoates with activated alkenes. In the presence of chiral phosphine **B2**, the [3 + 2] annulations between allenoates and 2-aryl-1,1-dicyanoethylenes allowed convenient syntheses of functionalized cyclopentenes with both aryl and heteroaryl substituents on the stereogenic carbon atom, in high yields and with up to 90% ee (Scheme 4) [37]. 3-Alkylideneindolin-2-ones underwent [3 + 2] annulations with allenoates, affording various biologically relevant spirocyclic oxindolic cyclopentanes in excellent yields and greater than 97% ee (Scheme 5) [38]. Enantioselective [3 + 2] annulations of 4-substituted 2,6-diarylidenecyclohexanones with allenoates occurred with high diastereo- and enantioselectivity, providing spirocyclic compounds in satisfactory yields with up to 92% ee (Scheme 6) [39]. Using the catalyst **B2**, Jørgensen and co-workers developed a sequential annulation/alcoholysis reaction. Alkylidene azlactones, among the most widely used starting materials for the syntheses of quaternary amino acids, were cyclized with ethyl allenoate and, subsequently, alcoholyzed in situ to afford highly functionalized, optically active amino esters in moderate to good yields and with 79–94% ee (Scheme 7) [40].

**Scheme 8:**
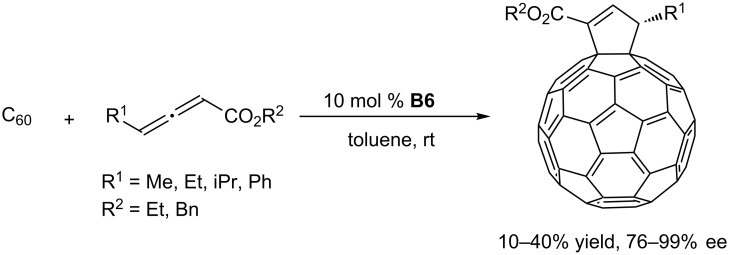
Asymmetric [3 + 2] annulations of C_60_ with allenoates, catalyzed by the chiral phosphine **B6**.

Very interestingly, when using the chiral phosphine (*S*,*S*)-*f*-binaphane **B6** as the catalyst, [60]fullerene also reacted with allenoates at room temperature, providing a wide range of optically pure (*S*)-cyclopenteno[60]fullerenes in up to 99% ee (Scheme 8) [41]. This study provided a versatile and promising strategy for tailoring carbon materials (e.g., fullerenes, carbon nanotubes), imparting them with desired properties for applications in materials chemistry [42–43].

**Scheme 9:**
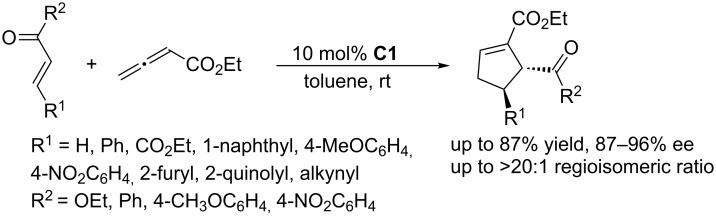
Asymmetric [3 + 2] annulations of α,β-unsaturated esters and ketones with an allenoate, catalyzed by the ferrocene-modified phosphine **C1**.

**Scheme 10:**
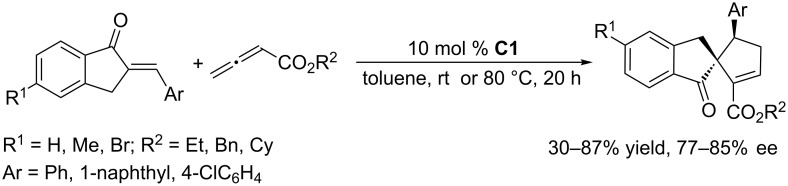
Asymmetric [3 + 2] annulations of exocyclic enones with allenoates, catalyzed by the ferrocene-modified phosphine **C1**.

**Scheme 11:**
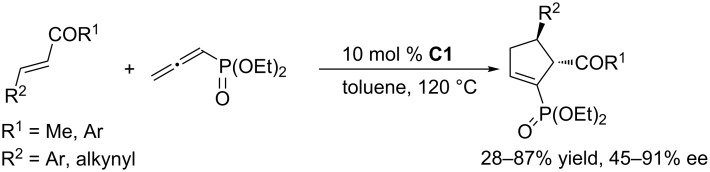
Asymmetric [3 + 2] annulations of enones with an allenylphosphonate, catalyzed by the ferrocene-modified phosphine **C1**.

**Scheme 12:**
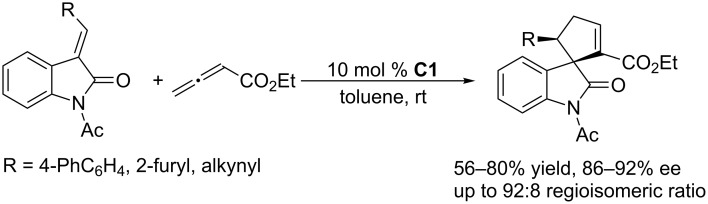
Asymmetric [3 + 2] annulations of 3-alkylidene-oxindoles with ethyl allenoate, catalyzed by the ferrocene-modified phosphine **C1**.

**Scheme 13:**
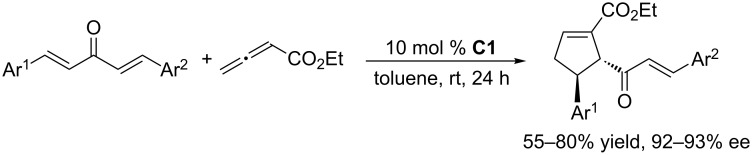
Asymmetric [3 + 2] annulations of dibenzylideneacetones with ethyl allenoate, catalyzed by the ferrocene-modified phosphine **C1**.

**Scheme 14:**
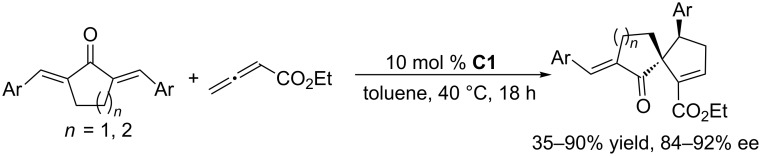
Asymmetric [3 + 2] annulations of trisubstituted alkenes with ethyl allenoate, catalyzed by the ferrocene-modified phosphine **C1**.

**Scheme 15:**
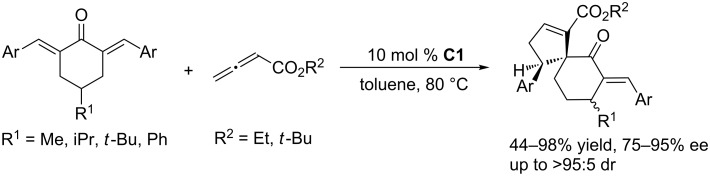
Asymmetric [3 + 2] annulations of 2,6-diarylidenecyclohexanones with allenoates, catalyzed by the ferrocene-modified phosphine **C1**.

**Scheme 16:**
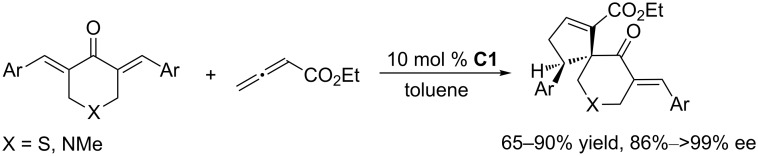
Asymmetric [3 + 2] annulations of α,β-unsaturated ketones with ethyl allenoates, catalyzed by the ferrocene-modified phosphine **C1**.

**Scheme 17:**
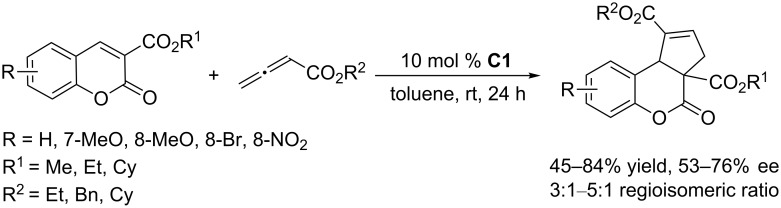
Asymmetric [3 + 2] annulations of α,β-unsaturated esters with allenoates, catalyzed by the ferrocene-modified phosphine **C1**.

To further develop nucleophilic phosphine-catalyzed asymmetric reactions, Marinetti and co-workers synthesized a series of ferrocene-modified planar chiral phosphines featuring a new skeleton (Figure 3) [44–45]. Among these compounds, the *P*-cyclohexyl phosphine **C1** proved to be the most efficient catalyst for [3 + 2] cycloadditions of ethyl 2,3-butadienoate with activated enones, fumarate esters, and acrylates. In the presence of 10 mol % of the catalyst in toluene at room temperature, the [3 + 2] annulations of allenoates with alkenes proceeded smoothly, providing functionalized cyclopentenes in moderate to good yields (up to 87%) with excellent enantioselectivities (87–96% ee) and regioisomeric ratios of up to >20:1 (Scheme 9) [44–45]. The bulky ferrocene was presumably responsible for the high enantioselectivities. Notably, the presence of the electron-rich ferrocene unit inhibited oxidation of the phosphines, imparting them with air-stability and easy-to-handle properties [45]. Subsequently, Marinetti and co-workers found that these chiral phosphines had very broad substrate scope and could be applied in [3 + 2] annulations of allenes with various activated alkenes. For example, the chiral phosphine **C1** mediated [3 + 2] annulations of a range of di- and trisubstituted alkenes with allenes under mild conditions, providing a variety of functionalized cyclopentenes, cyclopentenylphosphonates, spirooxindoles, heterocyclic spiranes, cyclopentene-fused chromanones, and dihydroquinolinones enantioselectively (Schemes 10–17) [38–39,46–48]. These products can be quite biologically active and many have been applied in medicine and other fields.

**Scheme 18:**
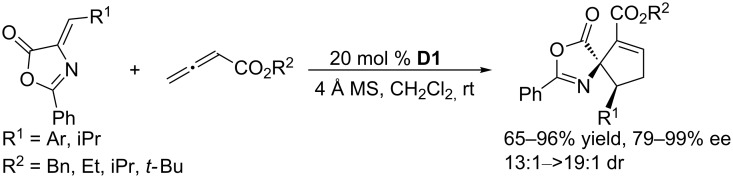
Asymmetric [3 + 2] annulations of alkylidene azlactones with allenoates, catalyzed by the chiral spiro phosphine **D1**.

Using the axially chiral spirophosphine **D1**, Shi and co-workers accomplished highly regioselective, diastereoselective, and enantioselective [3 + 2] annulations of a series of alkylidene azlactones with allenoates (Scheme 18) [49]. Under mild conditions, the reactions worked efficiently to afford corresponding functionalized spirocyclic products with adjacent spiro-quaternary and tertiary stereocenters in good to excellent yields. These products were readily transformed into a variety of useful optically active amino acid analogues, including various aspartic acid derivatives.

**Scheme 19:**
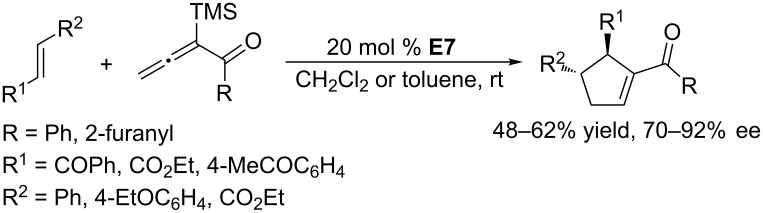
Asymmetric [3 + 2] annulations of α-trimethylsilyl allenones and electron-deficient olefins, catalyzed by the chiral phosphine **E7**.

Using the commercially available chiral catalyst (*S*,*S*)-Et-Duphos **E7**, Loh and co-workers developed the asymmetric [3 + 2] annulations of phenyl allenone and furanyl allenone with electron-deficient olefins, namely enones, maleates, and fumarates, to give corresponding functionalized cyclopentenes in moderate yields with moderate to high enantioselectivities (Scheme 19) [50]. The presence of a trimethylsilyl group at the α-position of the allenone was key to achieving a regioselective [3 + 2] annulation. This remarkable steric effect probably suppressed the [4 + 2] self-condensation of the allenone [51].

**Scheme 20:**
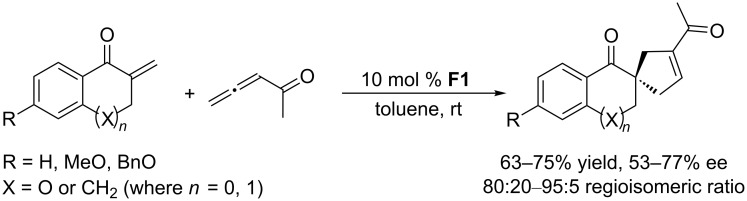
Asymmetric [3 + 2] annulations of α,β-unsaturated ketones with an allenone, catalyzed by the chiral phosphepine **F1**.

**2.1.2 [3 + 2] Annulations using acyclic phosphines as chiral catalysts:** In 2007, Wallace and co-workers employed the commercially available chiral phosphine (*S*,*S*)-DIOP **F1** in asymmetric [3 + 2] annulations of allenic ketones with a diverse array of exocyclic enones, providing a series of spirocyclic compounds – promising drug precursors – in good yields and with modest enantioselectivities (Scheme 20) [51]. Generally, it is difficult to control an enantioselective annulation when using an acyclic chiral phosphine lacking additional functionality. This example is one of the few asymmetric reactions catalyzed by an acyclic chiral phosphine.

**2.1.3 [3 + 2] Annulations using multifunctional phosphines as chiral catalysts:** In addition to cyclic chiral phosphines, multifunctional chiral phosphines can also display excellent catalytic activity and enantioselectivity in the asymmetric [3 + 2] annulations. Through intramolecular hydrogen bonding, the bond-forming transition-state geometry between electrophile and the zwitterionic intermediate formed from the allenoate and the multifunctional chiral phosphine can be better organized, thereby delivering annulation products in high yields and ee’s.

**Scheme 21:**
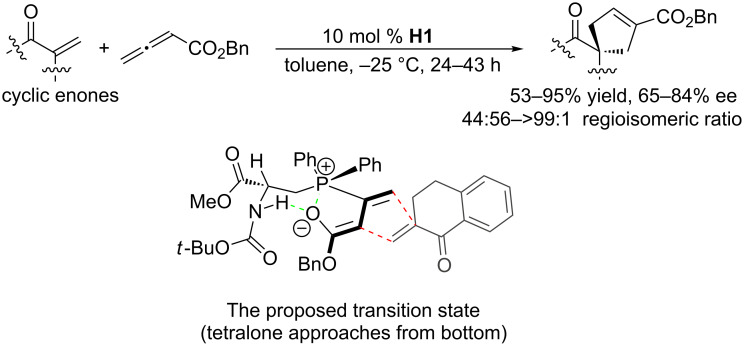
Asymmetric [3 + 2] annulations of cyclic enones with allenoates, catalyzed by the chiral α-amino acid-based phosphine **H1**, and the proposed transition state.

In 2007, based on their peptide catalyst studies, the Miller group developed the first α-amino acid-based phosphine catalyst **H1** for enantioselective [3 + 2] annulations of allenoates with enones (Scheme 21) [52]. This catalyst performed multiple roles during the catalytic process, with the amino acid moiety providing a chiral environment and acting as a hydrogen bond donor while the phosphine unit functioned as the nucleophile. In the presence of 10 mol % of **H1**, they treated both cyclic and acyclic enones with the allenoates in toluene at −25 °C to generate corresponding cyclopentenes as single regioisomers with high enantioselectivities. Interestingly, single amino acid-based phosphines were better than di-, tri-, and tetrapeptide-based catalysts. Of particular note, when they treated γ-substituted racemic allenoates with acyclic enones, unique dynamic kinetic asymmetric transformations occurred in the presence of a stoichiometric amount of the catalyst **H1**, giving highly substituted cycloadducts in excellent yields as single regio- and diastereoisomers with 87–93% ee. Although a catalytic amount of **H1** (20 mol %) also afforded 93% ee, the yield deteriorated to 38%. The proposed transition-state model (Scheme 21) illustrates how the dual control of activity and stereoselectivity was achieved: through formation of a zwitterionic intermediate from the allenoate and phosphine moiety and subsequent intramolecular hydrogen bonding between the NH unit and the oxygen atom that was formerly part of the allenoate’s carbonyl group.

**Scheme 22:**
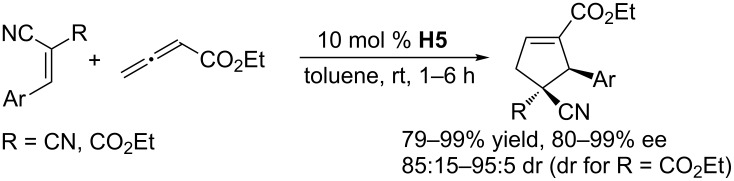
Asymmetric [3 + 2] annulations of arylidenemalononitriles and analogues with an allenoate, catalyzed by the chiral phosphine **H5**.

With the idea of using a natural amino acid as the hydrogen bonding framework, Zhao and co-workers designed the simpler multifunctional chiral *N*-acyl aminophosphine **H5**, which they readily synthesized in four steps from a commercially available Boc-protected amino alcohol [53]. With 10 mol % of catalyst **H5**, various arylidenemalononitriles reacted with an allenoate in toluene at room temperature for 1 h to provide various chiral cyclopentenes in 79–99% yield and 80–99% ee (Scheme 22). In particular, the annulations of 2-cyano-3-arylacrylates, with two different electron-withdrawing functional groups, produced chiral cyclopentenes bearing adjacent quaternary and tertiary stereocenters with exclusive regioselectivity and high diastereoselectivities and enantioselectivities. That study provided a significant advance in phosphine-catalyzed [3 + 2] annulations providing cyclopentenes. Notably, the use of PPh_3_ as the catalyst led to poor regioselectivities and moderate diastereoselectivities, revealing that the additional functional moiety was critical for accomplishing excellent enantioselectivity as well as regioselectivity and diastereoselectivity. A γ-substituted racemic allenoate also underwent the catalytic annulations smoothly through a dynamic kinetic asymmetric transformation, giving the desired products in high yields and moderate diastereoselectivities, albeit with somewhat decreased ee values. A transition-state model similar to Miller’s, mentioned above, was postulated.

**Scheme 23:**
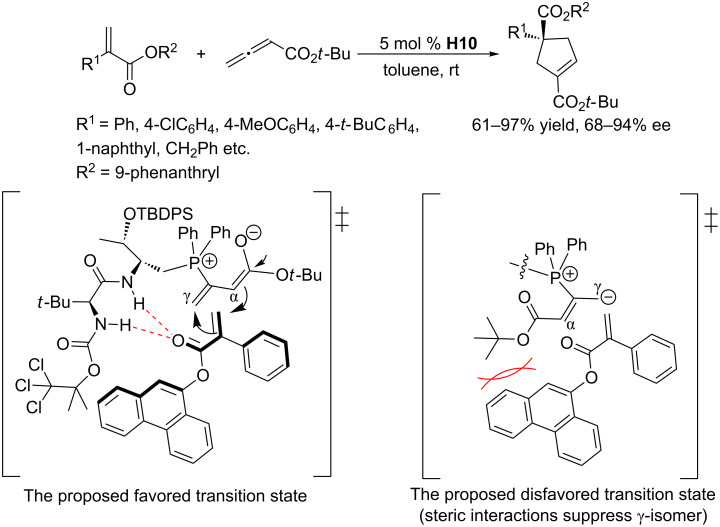
Asymmetric [3 + 2] annulations of α,β-unsaturated esters with an allenoate, catalyzed by the chiral phosphine **H10**, and possible transition states.

**Scheme 24:**
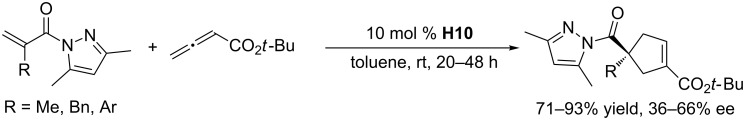
Asymmetric [3 + 2] annulations of 3,5-dimethyl-1*H*-pyrazole-derived acrylamides with an allenoate, catalyzed by the chiral phosphine **H10**.

**Scheme 25:**
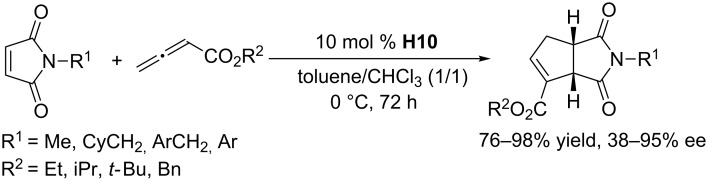
Asymmetric [3 + 2] annulations of maleimides with allenoates, catalyzed by the chiral phosphine **H10**.

Attracted by the phosphine-catalyzed reactions described above, Lu and co-workers prepared versatile dipeptide-derived phosphines for asymmetric annulations [54]. Their multifunctional catalyst framework comprised a dipeptide moiety and a tertiary phosphine unit. In the presence of 5 mol % of **H10** in toluene at room temperature, the asymmetric [3 + 2] annulations between allenoates and a series of activated olefins yielded exclusively the expected products in good to excellent yields and ee (Scheme 23) [54]. They proposed a transition state model to explain the stereoselectivity; steric hindrance between the *tert*-butyl group of the allenoate and the 9-phenanthryl group of the alkene suppressed the formation of the γ-isomer, affording α-adducts as the major regioselective products, with steric shielding of the *si*-face facilitating production of the major enantiomer (Scheme 23). Shortly after, the Lu group further expanded the substrate scope of asymmetric [3 + 2] annulations with allenoates to a series of 3,5-dimethyl-1*H*-pyrazole-derived acrylamides (Scheme 24) [55]. The dipeptide-based phosphine **H10** effectively promoted the reaction in good to excellent yields, albeit low to moderate enantioselectivities. In 2012, Lu, Shi, and co-workers found that the dipeptide-based phosphine **H10** was also quite effective as a chiral catalyst for [3 + 2] annulations of various maleimides with allenoates (Scheme 25) [56]. They obtained a wide range of bicyclic cyclopentenes in good to excellent yields. *N*-Alkyl-substituted maleimides were converted to cyclopentenes in high ee, while *N-*aryl-substituted maleimides underwent the reaction with low to moderate enantioselectivity.

**Scheme 26:**
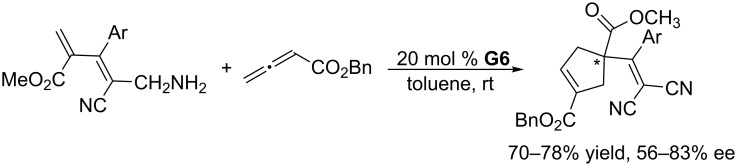
Asymmetric [3 + 2] annulations of α-substituted acrylates with allenoate, catalyzed by the chiral phosphine **G6**.

Using the multifunctional chiral phosphine **G6**, featuring a binaphthyl skeleton and bearing a thiourea moiety, Shi and co-workers developed an asymmetric [3 + 2] annulation of α-substituted acrylates with an allenoate (Scheme 26) [57]. The reactions proceeded smoothly in toluene at room temperature to give the corresponding functionalized cyclopentenes in high yields with moderate to good ee. The substrate scope was, however, very limited.

#### 2.2 [3 + 2] Annulations of allenes with activated imines

Phosphine-catalyzed [3 + 2] annulation of allenes with activated imines has emerged as an important tool for the synthesis of functionalized pyrrolines, which are valuable heterocyclic compounds for the synthesis of bioactive compounds and natural products. At the end of the century, Lu and co-workers discovered the nucleophilic phosphine-catalyzed [3 + 2] annulation of allenes with electron-deficient imines and established a reasonable reaction mechanism [58–60]. Its asymmetric version, however, did not receive any attention for almost 10 years.

**Scheme 27:**
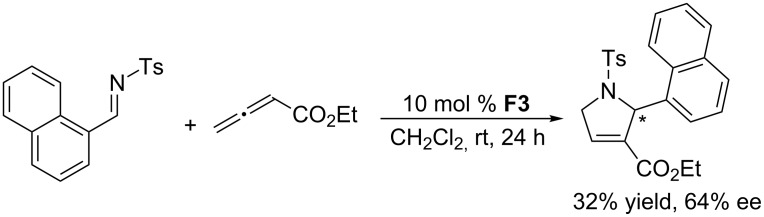
Asymmetric [3 + 2] annulation of an *N*-tosylimine with an allenoate, catalyzed by the chiral phosphine **F3**.

**Scheme 28:**
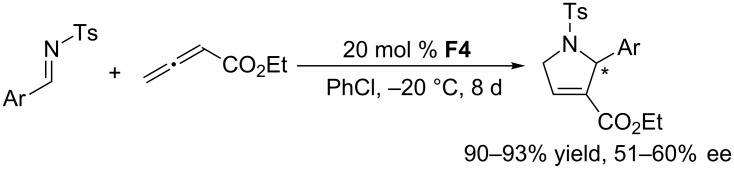
Asymmetric [3 + 2] annulations of *N*-tosylimines with an allenoate, catalyzed by the chiral phosphine **F4**.

In 2006, Marinetti and co-workers reported the first asymmetric [3 + 2] annulations of imines with allenoates [61]. In this initial exploration, they screened various cyclic and acyclic chiral phosphines, finding that 10 mol % of the acyclic chiral phosphine (*S*)-PHANEPHOS **F3** provided the corresponding pyrroline (Scheme 27) in comparatively high ee (64%), albeit in low yield (32%). Other cyclic and acyclic chiral phosphines provided generally low enantioselectivities. In the same year, Gladysz and Scherer investigated the behavior of an interesting chiral rhenium-containing phosphine **F4** in the asymmetric [3 + 2] annulation of allenoates with *N*-tosylimines (Scheme 28) [62]. Gratifyingly, this acyclic chiral phosphine could efficiently catalyze this transformation, providing pyrroline derivatives in excellent yields (90–93%), albeit after long reaction times (8 days) and with moderate ee (51–60%).

**Scheme 29:**
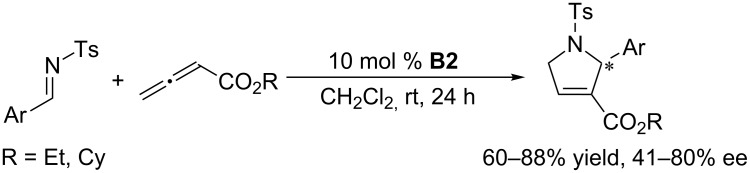
Asymmetric [3 + 2] annulations of *N*-tosylimines with an allenoate, catalyzed by the chiral phosphine **B2**.

**Scheme 30:**
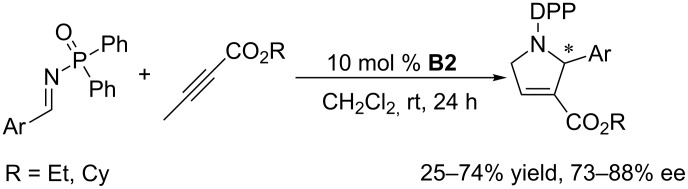
Asymmetric [3 + 2] annulations of *N*-diphenylphosphinoyl aromatic imines with butynoates, catalyzed by the chiral phosphine **B2**.

**Scheme 31:**
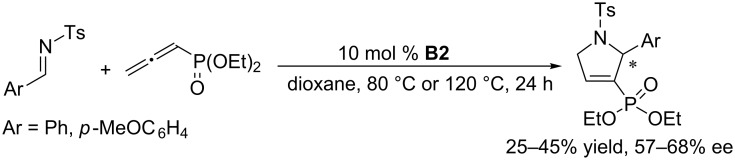
Asymmetric [3 + 2] annulations of *N*-tosylimines with allenylphosphonates, catalyzed by the chiral phosphine **B2**.

Marinetti et al. employed the binaphthyl-based chiral cyclic phosphine **B2** to improve the enantioselectivities of the [3 + 2] annulation of allenoates with *N*-tosylimines. Compared with the performance of acyclic chiral phosphines, the results were indeed improved, obtaining pyrroline products in 41–80% ee, although the enantioselectivities remained unsatisfactory (Scheme 29) [63]. In subsequent investigations of asymmetric [3 + 2] annulations performed with *N*-diphenylphosphinoylimines (Scheme 30) and allenylphosphonates (Scheme 31) as substrates [64–65], the former reactions generated pyrrolines with good ee (73–88%), albeit with moderate yields (25–74%) [64]. The relative ease of removal of the diphenylphosphinoyl (DPP) protecting group makes this reaction quite valuable as an organic transformation for the preparation of secondary pyrrolines. The latter reactions (Scheme 31) required harsh conditions, leading to pyrroline derivatives in low yields with moderate ee [65]. In terms of both conversion and enantioselectivity, binaphthyl skeleton-based cyclic chiral phosphines are not ideal catalysts for asymmetric [3 + 2] annulations of electron-deficient imines with allenes.

**Scheme 32:**
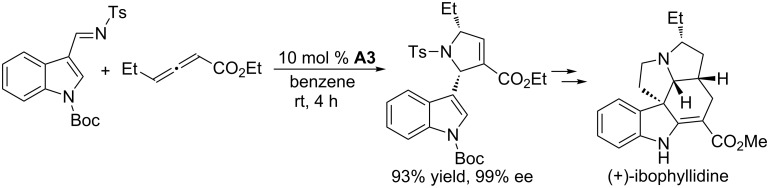
Asymmetric [3 + 2] annulation of an *N*-tosylimine with an allenoate, catalyzed by the chiral phosphine **A3**, and its application in the total synthesis of (+)-ibophyllidine.

On the other hand, the rigid bridged [2.2.1] bicyclic chiral phosphine **A3** appears to be an excellent catalyst for allene/imine [3 + 2] annulation. With a chiral phosphine-catalyzed [3 + 2] annulation of an indole-derived imine and an γ-ethylallenoate as the key step, Kwon and Andrews completed the first enantioselective total synthesis of the indole alkaloid (+)-ibophyllidine in 15 steps and 13% overall yield from *N*-Boc-indole-3-aldehyde (Scheme 32) [66]. This approach was the first non-formal total synthesis of a complex natural product employing phosphine-catalyzed asymmetric [3 + 2] annulation. In the key transformation, using 10 mol % of the P-chiral [2.2.1]bicyclic phosphine **A3** derived from *trans*-L-4-hydroxyproline, asymmetric [3 + 2] annulation of 4-ethyl-2,3-butadienoate with an *N*-tosylaldimine (prepared in 90% yield through condensation of *p*-toluenesulfonamide with *N*-Boc-indole-3-carbaldehyde) for 4 h at room temperature proceeded exceedingly well, giving the desired pyrroline in 93% yield with 99% ee and high diastereoselectivity. In particular, the reaction could be performed on multigram scale to provide the optically pure pyrroline; indeed, in the presence of 10 mol % of the catalyst, the annulation performed on an approximately 30 g scale proceeded in 94% yield and 97% ee.

**Scheme 33:**
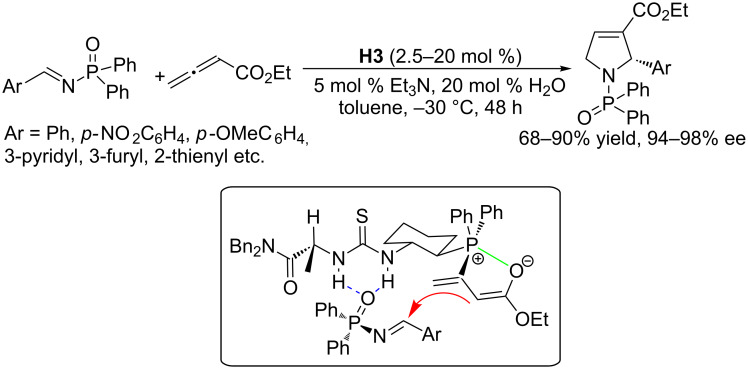
Asymmetric [3 + 2] annulations of *N*-diphenylphosphinoyl aromatic imines with allenoates (top), catalyzed by the chiral phosphine **H3**, and a possible transition state (bottom).

By using multifunctional chiral phosphines, Jacobsen and Fang obtained a major breakthrough in development of highly enantioselective [3 + 2] annulations of allenoates with imines. Based on thiourea-based catalyst systems, they developed the multifunctional catalyst **H3** containing a phosphine fragment, a thiourea moiety, and an amino acid residue [67]. In the presence of 10 mol % of catalyst **H3**, with the assistance of triethylamine (5 mol %) and water (20 mol %), a wide range of *N*-diphenylphosphinoyl aromatic imines underwent cyclizations (Scheme 33) with an allenoate in toluene at −25 °C for 48 h, affording dihydropyrrole derivatives in 68–90% yield and excellent ee (94–98%). The catalytic amounts of triethylamine and water significantly increased the reaction rates, facilitating proton transfer and catalyst regeneration in the reaction process. In the case of *o*-bromophenylimine, even 2.5 mol % of **H3** could deliver the corresponding product without any loss of enantioselectivity (95% ee), albeit in slightly lower yield. Jacobsen and Fang proposed a possible transition state to explain the high enantioselectivity. In this cooperative system, the phosphine moiety was responsible for activation of the allenoate and the enantioinduction, while the thiourea unit played the dual roles of activating the imine and stereochemically controlling the association of the phosphoryl substituents of the imine. Although the accomplishments with this thiourea-based chiral catalyst were unprecedented, the substrate scope of this reaction is restricted to diphenylphosphinoyl aromatic imines because aliphatic imines decompose under the optimal conditions.

**Scheme 34:**
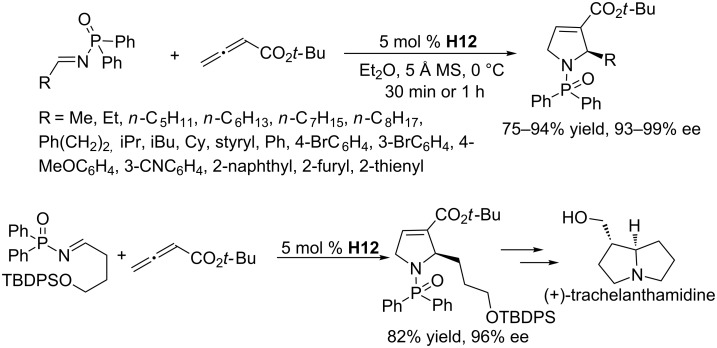
Asymmetric [3 + 2] annulation of *N*-diphenylphosphinoylimines with allenoates, catalyzed by the chiral phosphine **H12**, and its application in the formal synthesis of (+)-trachelanthamidine.

To overcome the limited substrate scope, Lu and co-workers explored imine–allene [3 + 2] annulations catalyzed by a dipeptide-derived phosphine [68]. They identified the phosphine **H12**, a close analogue of **H10**, as the most suitable catalyst. In the presence of 5 mol % of **H12**, a wide range of alkylimines and various arylimines could be employed as reaction partners. The [3 + 2] annulations of allenoates with *N*-diphenylphosphinoylimines proceeded smoothly in ethyl ether in the presence of 5 Å molecular sieves at 0 °C within 30–60 min, providing a variety of pyrroline derivatives in high yields with uniformly excellent enantioselectivities (Scheme 34). Chiral 2-alkyl-substituted 3-pyrrolines are highly valuable building blocks that can be further transformed into various biologically useful molecules. For example, with this reaction as a key step, a concise formal synthesis of (+)-trachelanthamidine was accomplished, highlighting the synthetic value of this methodology (Scheme 34).

#### 2.3 [3 + 2] Annulations of allenes with azomethine imines

**Scheme 35:**
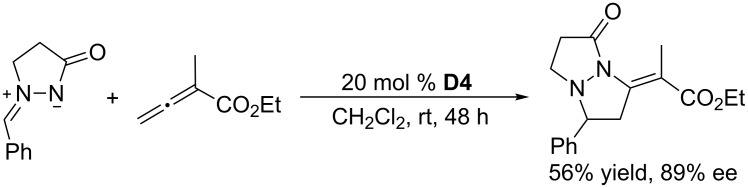
Asymmetric [3 + 2] annulation of an azomethine imine with an allenoate, catalyzed by the chiral phosphine **D4**.

Using the chiral catalyst **D4**, a cyclic phosphine featuring a spiro-skeleton, Guo, Kwon, and co-workers achieved asymmetric [3 + 2] annulation of an allenoate with an azomethine imine (Scheme 35) [69]. The reaction performed in dichloromethane at room temperature for 48 h afforded the product tetrahydropyrazolopyrazolone in 56% yield and 89% ee. Although only a single example was reported, their paper demonstrated that enantioselective [3 + 2] annulations of allenoates with azomethine imines could be accomplished when using a suitable chiral catalyst.

#### 2.4 [3 + 2] Annulations of alkynes with activated alkenes

**Scheme 36:**
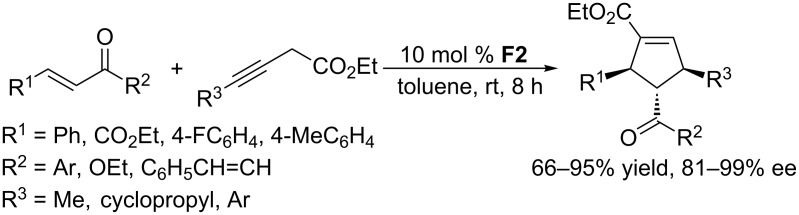
Asymmetric [3 + 2] annulations between α,β-unsaturated esters/ketones and 3-butynoates, catalyzed by the chiral phosphine **F2**.

In addition to allenoates, alkynes are also compatible substrates for asymmetric [3 + 2] annulations with activated alkenes. Using the commercially available chiral catalyst (*R*,*R*)-DIPAMP **F2**, 3-butynoates underwent [3 + 2] annulations with electron-deficient olefins, providing highly functionalized cyclopentenes in 66–95% yield with 81–99% ee (Scheme 36) [70]. Interestingly, this acyclic catalyst proved to be remarkably efficient at mediating these tandem reactions, despite its structure being less rigid than most of aforementioned cyclic chiral phosphines. Control experiments indicated that under phosphine catalysis conditions, 3-butynoate initially isomerized to the allenoate, which subsequently underwent [3 + 2] annulations with activated alkenes. Notably, only 10 mol % of catalyst **F2** was required, even though it was responsible for promoting the two proposed steps.

#### 2.5 [3 + 2] Annulations of MBH carbonates with activated alkenes

MBH carbonates, which are readily accessible from the adducts of MBH reactions, have been used extensively in organocatalysis for the formation of C–C and C–heteroatom bonds [71–72]. In addition to activated allenes and alkynes, MBH carbonates are often employed as versatile substrates for the phosphine-catalyzed annulations.

**Scheme 37:**
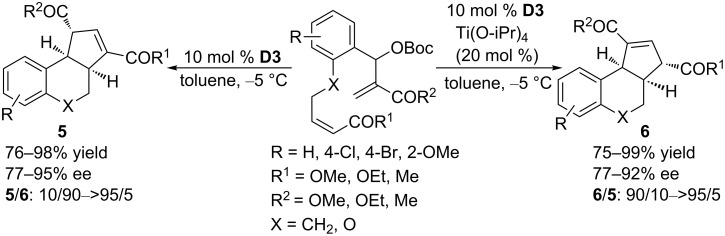
Asymmetric intramolecular [3 + 2] annulations of electron-deficient alkenes and MBH carbonates, catalyzed by the chiral phosphine **D3**.

In 2010, using the cyclic phosphine (*S*)-DMM-SITCP **D3**, Tang and Zhou developed an intramolecular asymmetric [3 + 2] annulation by combining MBH carbonates and electron-deficient alkenes into a single molecule (Scheme 37) [73]. In the presence of 10 mol % of **D3**, a variety of α,β-unsaturated carbonyl compounds were transformed efficiently in toluene at −5 °C to give optically active benzobicyclo[4.3.0] compounds **5** in excellent yields with high enantioselectivities (77–95% ee). Interestingly, the addition of 20 mol % of Ti(O-iPr)_4_, under otherwise identical conditions, inhibited the isomerization process, causing the benzobicyclo[4.3.0] compounds **6** to be obtained as major products in excellent yields with high enantioselectivities (77–92% ee).

**Scheme 38:**
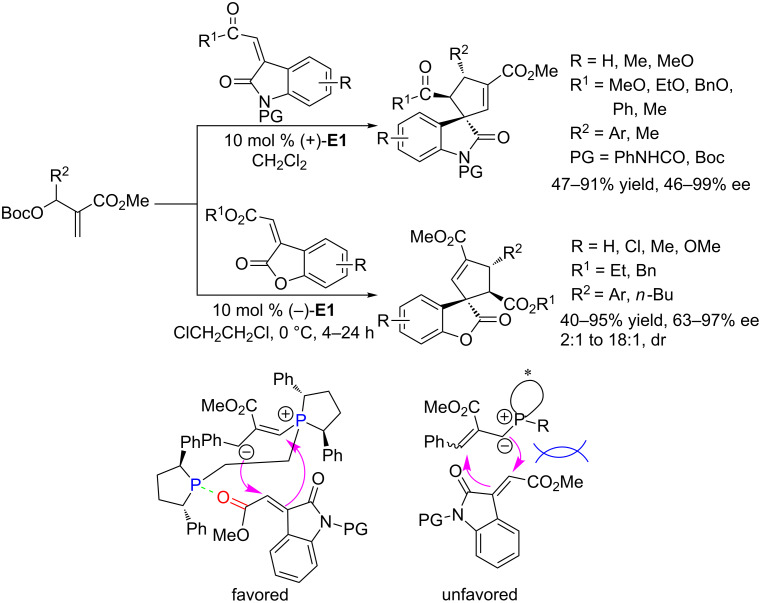
Asymmetric [3 + 2] annulations of methyleneindolinone and methylenebenzofuranone derivatives with MHB carbonates, catalyzed by the chiral phosphines (+)-**E1** and (–)-**E1**, and possible transition states.

In 2011, using the chiral phosphine (+)-Ph-BPE **E1** as the catalyst, Barbas and co-workers achieved asymmetric [3 + 2] annulations of MBH carbonates with methyleneindolinones (Scheme 38) [74]. In the presence of 10 mol % of this catalyst, the reaction proceeded well in dichloromethane to furnish a wide range of spirocyclopenteneoxindoles in moderate to excellent yields and ee's (Scheme 38). The yields and stereoselectivities were significantly influenced not by electronic effects but by aromatic interactions. Aryl-substituted MBH carbonates reacted smoothly to afford polyfunctionalized spirocyclopenteneoxindoles in good yields (63–85%) and with excellent enantioselectivities (91–99% ee). In contrast, the ee was fairly low (46%) when using a methyl-substituted MHB carbonate at the allylic site. It was rationalized that the aromatic π–π interactions between the catalyst and the substrates favored the formation of the products. Based on control experiments and aforementioned results, Barbas and co-workers proposed a plausible mechanism and suggested that the high stereoselectivity resulted from steric interactions between the bulky substituent of the phosphonium ylide from the MBH carbonate and the carboxylic ester of methyleneindolinone shielding one possible attacking face during the nucleophilic attack (Scheme 38). In 2013, using the same catalytic system, Barbas and co-workers further developed highly stereoselective phosphine-catalyzed [3 + 2] annulations of MBH carbonates with methylenebenzofuranone derivatives, constructing a variety of complex polysubstituted spirocyclopentenebenzofuranones in high yields with good to excellent enantioselectivities [75].

**Scheme 39:**
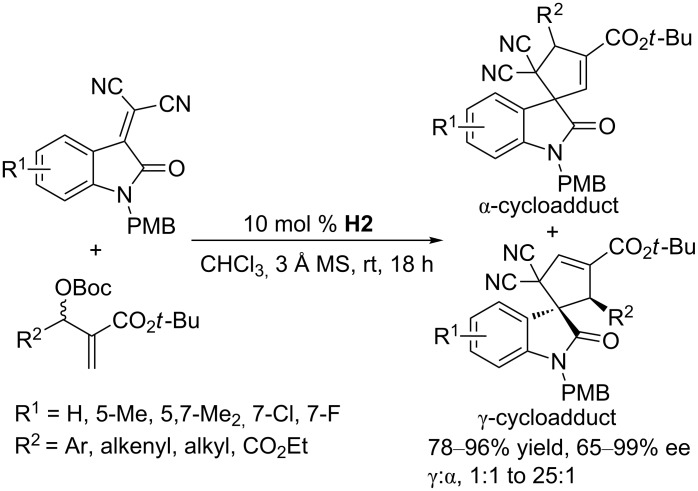
Asymmetric [3 + 2] annulations of activated isatin-based alkenes with MBH carbonates, catalyzed by the chiral phosphine **H2**.

Using the amino acid-derived chiral phosphine **H2**, Lu and co-workers explored asymmetric [3 + 2] cycloadditions between MBH carbonates and activated isatin-based alkenes (Scheme 39) [76]. The reactions, performed in chloroform in the presence of molecular sieves at room temperature, provided biologically important 3-spirocyclopentene-2-oxindoles with two contiguous quaternary centers in very high yields and with good enantioselectivities; they tolerated a wide range of MBH carbonates featuring different electronic properties for their aromatic and heteroaromatic moieties at the allylic position (e.g., phenyl, 4-cyanophenyl, 4-methylphenyl, 2-naphthyl, 3-furyl) as well as diverse isatin-derived alkenes having substituents on their phenyl rings. Of particular interest, even the relatively inert MBH adduct presenting an alkyl unit at the allylic position was applicable to the reactions, furnishing the corresponding product in good yield and enantioselectivity, albeit lower regioselectivity (1:2). After studying the substrate scope and limitations, Lu and co-workers proved that these reactions could be performed in a convenient one-pot manner. For example, the one-pot reactions of isatins, malononitriles (precursors of activated alkene), and MBH adducts produced corresponding spirooxindoles with the same enantioselectivity as that between activated alkenes and MBH adducts, albeit in slightly diminished yields. Shi and co-workers also studied this reaction, but using the chiral bifunctional thiourea-phosphine catalyst **G7**. The [3 + 2] annulation of MBH carbonate with an activated isatin-based alkene in toluene at room temperature gave the corresponding γ-cycloadduct as the major product in 92% yield, with 9:1 dr and 74% ee [77].

**Scheme 40:**
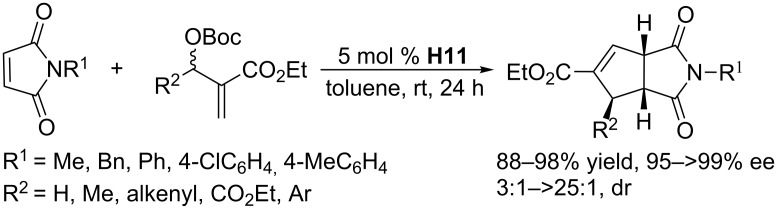
Asymmetric [3 + 2] annulations of maleimides with MBH carbonates, catalyzed by the chiral phosphine **H11**.

Using the amino acid-derived chiral phosphine catalyst **H11**, Lu and co-workers performed asymmetric [3 + 2] annulations between MBH carbonates and maleimides, obtaining access to a wide range of bicyclic imides in excellent yields and enantioselectivities and high diastereoselectivities (Scheme 40) [78]. This methodology worked very well even when operated on gram-scale.

**Scheme 41:**
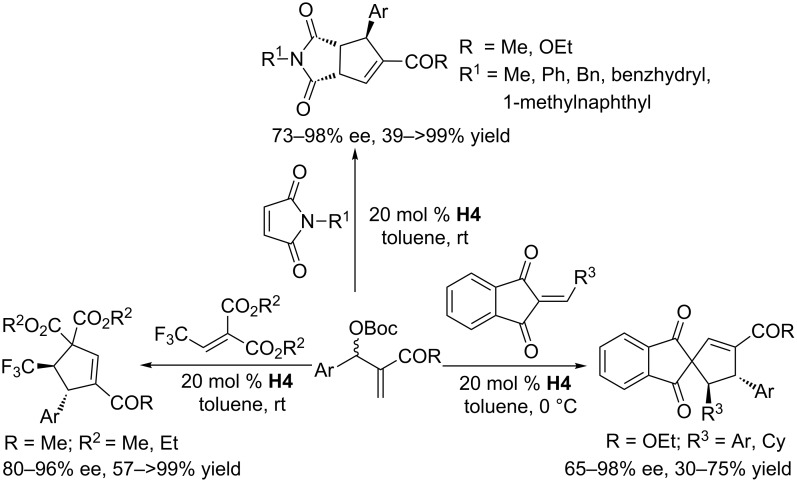
A series of [3 + 2] annulations of various activated alkenes with MBH carbonates, catalyzed by the chiral phosphine **H4**.

In 2012, using multifunctional chiral phosphines as catalysts, Shi and co-workers investigated a range of asymmetric [3 + 2] annulations of MBH carbonates with various activated alkenes (Scheme 41). They synthesized a range of multifunctional thiourea-phosphines, among which the chiral phosphine **H4** proved to be a versatile and powerful catalyst for these asymmetric [3 + 2] annulations. In the presence of 20 mol % of this chiral phosphine, various activated alkenes, including maleimides [79], trifluoroethylidenemalonate [80], and 2-arylideneindane-1,3-diones [81], were highly compatible for asymmetric [3 + 2] annulation reactions with MBH carbonates, providing moderate to excellent yields, diastereoselectivities, and enantioselectivities (Scheme 41). An array of substituted MBH carbonates bearing neutral, electron-withdrawing, and electron-donating aromatic groups were effectively converted to the corresponding functionalized cyclopentenes. Nevertheless, MBH carbonates having aliphatic substituents, rather than aromatic ones, at the allylic position were not tolerated in these transformations.

#### 2.6 [3 + 2] Annulations of an alkyne with isatins

**Scheme 42:**
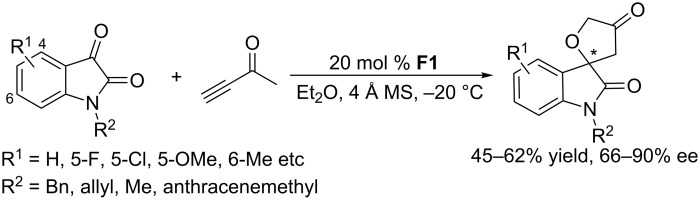
Asymmetric [3 + 2] annulations of an alkyne with isatins, catalyzed by the chiral phosphine **F1**.

Using the acyclic chiral phosphine (4*S*,5*S*)-DIOP **F1**, Shi and co-workers developed asymmetric [3 + 2] annulations of but-3-yn-2-one with N-protected isatins (Scheme 42) [82]. In the presence of 20 mol % of **F1**, but-3-yn-2-one reacted with a series of N-protected isatins in ethyl ether at −20 °C to afford enantioenriched spiro[furan-2,3´-indoline]-2´,4(5*H*)-diones with good to excellent ee's, albeit moderate yields.

#### 2.7 [4 + 2] Annulations of allenes with activated imines

**Scheme 43:**
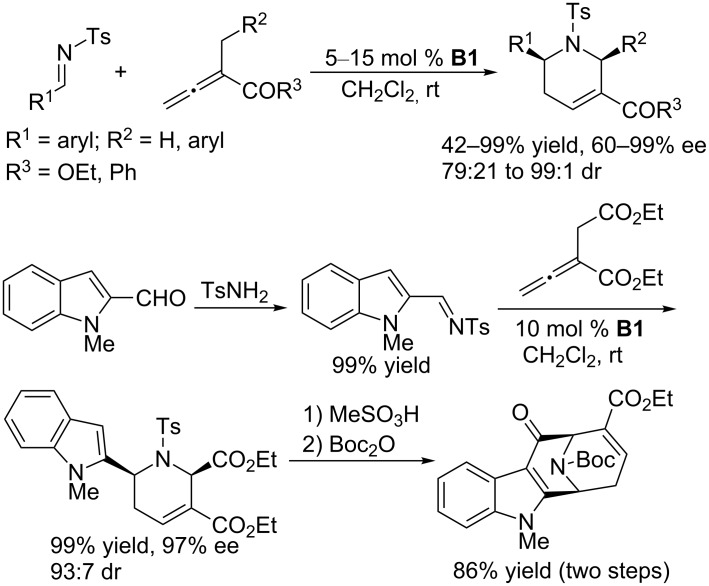
Asymmetric [4 + 2] annulations catalyzed by the chiral phosphine **B1**.

Compared with phosphine-catalyzed asymmetric [3 + 2] annulations, asymmetric [4 + 2] annulations have been less studied, with only limited examples reported. In 2005, based on Kwon’s phosphine-catalyzed [4 + 2] annulation of allenoates with *N*-tosylimines [83], Fu and co-workers developed an asymmetric variant using the binaphthyl-based chiral cyclic phosphine **B1** (Scheme 43) [84]. In the presence of 5 mol % of **B1**, asymmetric [4 + 2] annulations of allenoates with a broad range of aromatic *N*-tosylimines worked efficiently in dichloromethane at room temperature to give an array of chiral piperidine derivatives in good to excellent stereoselectivities (up to 99% ee, up to 99:1 dr) and moderate to excellent yields (42–99%). The piperidine products could be transformed conveniently into biologically important heterocyclic compounds. For example, with this asymmetric [4 + 2] annulation as the key step, using indole-2-carboxaldehyde as the starting material, the bridged tetracyclic framework of the *Alstonia* class of indole alkaloids was readily formed in high yield. This asymmetric [4 + 2] annulation was a seminal advance in the area of nucleophilic phosphine catalysis, attracting much attention toward chiral phosphine-catalyzed asymmetric reactions.

**Scheme 44:**
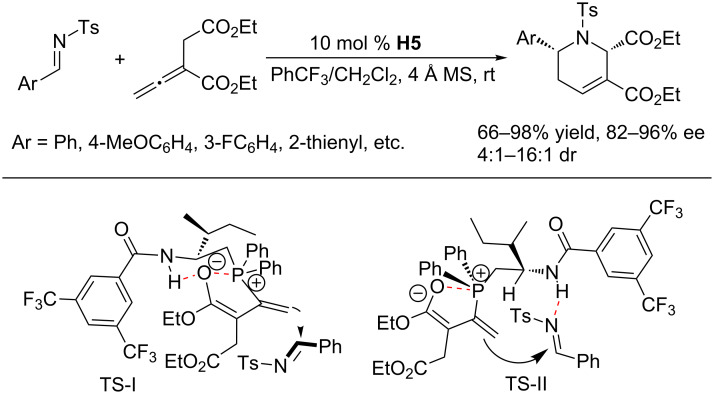
Asymmetric [4 + 2] annulations catalyzed by the chiral phosphine **H5**.

Using the bifunctional *N*-acylaminophosphine **H5**, Zhao and co-workers also achieved enantioselective [4 + 2] annulations of allenoates with *N*-tosylaldimines (Scheme 44) [85]. In the presence of 4 Å molecular sieves and with PhCF_3_ and CH_2_Cl_2_ as the mixed solvent, **H5**-catalzyed enantioselective [4 + 2] annulations between α-substituted allenoates and *N*-tosylaldimines afforded a wide range of tetrahydropyridines in high yields and with good to excellent enantioselectivities. For some imines, the bifunctional phosphine displayed catalytic capability superior to that of Fu’s monophosphine system [84] in terms of yield, ee, or both. Based on previous literature, Zhao and co-workers speculated two possible transition states; in both cases, the dienolate adopts a conformation, featuring hydrogen bonding and O–P electrostatic attraction, that favors *re*-face attack of the imine (Scheme 44).

#### 2.8 [4 + 2] Annulations of allenes with activated alkenes

**Scheme 45:**
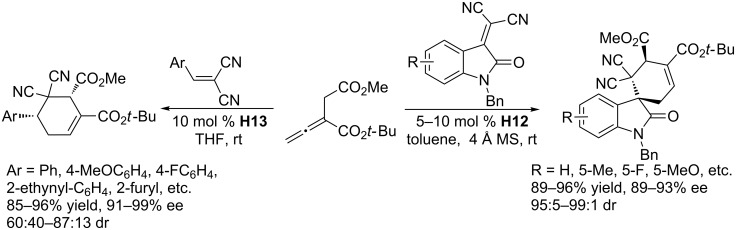
Asymmetric [4 + 2] annulations catalyzed by the chiral phosphines **H13** and **H12**.

Phosphine-catalyzed [4 + 2] annulation of an allenoate with an activated alkene is a powerful tool for the synthesis of a functionalized cyclohexene [86–87]. To develop an asymmetric version, Lu and co-workers screened 14 bifunctional chiral amino acid-derived phosphines, from which they found that the amido phosphine **H13** was the most efficient catalyst for the [4 + 2] annulations of allenoates with 2-aryl-1,1-dicyanoethylenes (Scheme 45) [88]. In the presence of 10 mol % of **H13** in THF at room temperature, the 1,1-dicyanoethylenes reacted with the α-substituted allenoates to afford an array of functionalized cyclohexenes. A wide range of 2-aryl- and 2-heteroaryl-1,1-dicyanoethylenes were applicable to this transformation, rendering high yields, moderate to good diastereoselectivities, and excellent enantioselectivities. These reaction conditions were not applicable, however, to transformations involving isatin-derived alkenes as substrates, resulting in very poor diastereoselectivities and enantioselectivities. For these oxindole derivatives, the use of the chiral dipeptide-derived phosphine **H12** in toluene at room temperature provided the corresponding [4 + 2] cycloadducts, 3-spirocyclohexene-2-oxindoles, in high yields with excellent ee and dr. These asymmetric [4 + 2] annulations of allenoates with activated alkenes significantly offset the limitations of Diels–Alder reactions when synthesizing enantioenriched multisubstituted cyclohexenes.

**Scheme 46:**
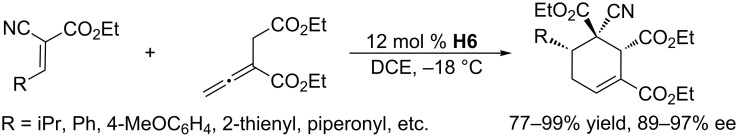
Asymmetric [4 + 2] annulations catalyzed by the chiral phosphine **H6**.

At about the same time, Zhao and co-workers also examined the application of bifunctional chiral phosphines in asymmetric [4 + 2] annulations between activated alkenes and α-substituted allenoates (Scheme 46) [89]. They found that the chiral *N*-acyl aminophosphine **H6** in 1,2-dichloroethane at −18 °C mediated a smooth reaction to produce various optically active cyclohexenes containing three neighboring carbon stereocenters in high yields and with excellent ee. Notably, not only arylidenecyanoacetates but also alkylidenecyanoacetates (e.g., isobutylidenecyanoacetate) were applicable to the reaction, being converted to their desired products in excellent yields and ee.

#### 2.9 [2 + 2] Annulations of ketenes with imines

**Scheme 47:**
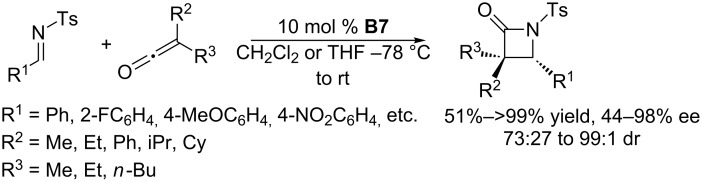
Kerrigan’s [2 + 2] annulations of ketenes with imines, catalyzed by the chiral phosphine **B7**.

Various ketenes, another class of cumulenes that are analogues of allenes, are also suitable substrates for phosphine-catalyzed asymmetric annulations. Unlike allenes, which act as three- and four-carbon synthons, ketenes typically serve as binary synthons in their annulations. Recently, using the cyclic chiral phosphine (*R*)-BINAPHANE **B7**, the Kerrigan group developed phosphine-catalyzed asymmetric [2 + 2] annulations of ketenes with imines (Scheme 47) [90]. In the presence of 10 mol % of **B7** in dichloromethane or tetrahydrofuran, the reaction of disubstituted ketenes and *N*-tosyl arylimines provided corresponding *trans*-β-lactams in moderate to excellent ee (up to 98%), diastereoselectivities (up to 99:1 dr), and yields (up to >99%). Of particular interest, this methodology facilitates the formation of diverse *trans*-β-lactams that are complimentary to the related *cis*-lactams.

#### 2.10 [4 + 1] Annulations of MBH carbonates with dienes

**Scheme 48:**
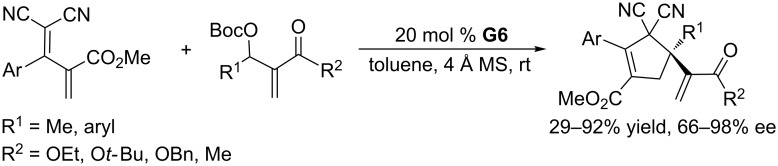
Asymmetric [4 + 1] annulations, catalyzed by the chiral phosphine **G6**.

Phosphine-catalyzed asymmetric [4 + 1] annulations between MBH carbonates and activated dienes could be achieved when using the bifunctional phosphine **G6** as the catalyst (Scheme 48) [91]. The reaction worked efficiently in the presence of 4 Å molecular sieves in toluene at room temperature to furnish a variety of functionalized cyclopentenes bearing quaternary carbon stereocenters in 29–92% yield with 66–98% ee. Unfortunately, the reaction times were long (up to 7 days), and substrates with bulky substituents were not well tolerated, giving low yields or ee. Notably, the reactions proved that MBH carbonates can serve as one-carbon synthons for the annulations and expanded the utility of MBH carbonates in synthetic chemistry.

#### 2.11 Annulations through homodimerization of ketoketenes

**Scheme 49:**
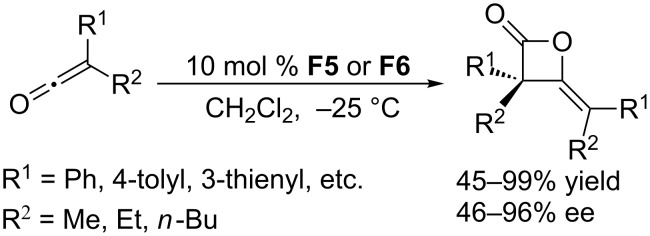
Asymmetric homodimerization of ketenes, catalyzed by the chiral phosphine **F5** and **F6**.

Using the chiral catalysts (*S*,*R*p)-Josiphos **F5** and **F6**, the Kerrigan group developed the asymmetric homodimerization of ketenes (Scheme 49) [92]. This self-condensation of ketoketenes proceeded in dichloromethane at –25 °C to give chiral β-lactones in high yields (up to 99%) with good to excellent ee (up to 96%). In subsequent transformations, the β-lactone products underwent various ring opening reactions to provide very useful derivatives, such as 1,3-diketones and enol esters, with good diastereoselectivity.

#### 2.12 Annulations through domino aza-MBH/Michael reactions

**Scheme 50:**
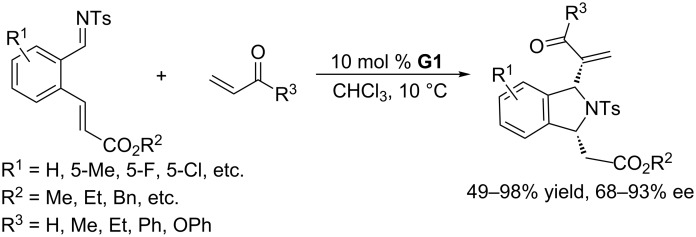
Aza-MBH/Michael reactions, catalyzed by the chiral phosphine **G1**.

Because organocatalytic asymmetric domino reactions allow the rapid construction of structurally complex molecules from readily available starting materials in two or more steps in a single operation, they have attracted much attention. Bifunctional chiral phosphines are ideal chiral catalysts for tandem reactions. In 2010, using the bifunctional chiral phosphine **G1**, bearing both Brønsted acid and Lewis base units, as the catalyst, asymmetric domino aza-MBH/aza-Michael reactions of activated alkenes and *N*-tosylimines with Michael acceptor moieties at their ortho positions were accomplished to give chiral 1,3-disubstituted isoindolines (Scheme 50) [93]. Good to excellent yields were obtained with excellent ee. A wide range of activated alkenes containing acyl, alkoxycarbonyl, and formyl groups and a series of *N*-tosylarylimines with alkyl- and halo-substituted benzene rings were compatible with the reaction conditions. The obtained products could be transformed smoothly into many complex and potentially useful compounds, through diverse derivatization, without significant losses of enantiomeric excesses.

#### 2.13 Annulations through tandem RC/Michael reactions

**Scheme 51:**
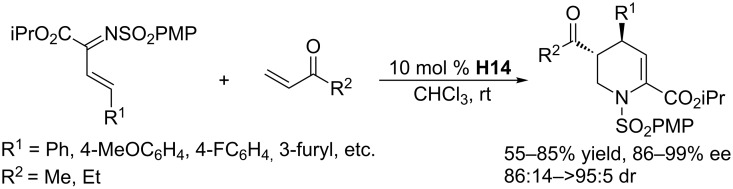
Tandem RC/Michael additions, catalyzed by the chiral phosphine **H14**.

In 2012, using the chiral threonine-derived phosphine **H14**, Zhong and Loh developed asymmetric [4 + 2] annulations of activated *N*-sulfonyl-1-aza-1,3-dienes and alkenes through tandem RC/Michael reactions (Scheme 51) [94]. In the presence of 10 mol % of **H14**, various 1-aza-1,3-dienes smoothly underwent [4 + 2] annulations with enones in chloroform at room temperature, affording a broad spectrum of densely functionalized tetrahydropyridine derivatives, with exclusive 4,5-*trans* diastereoselectivity, excellent enantioselectivity, and good to excellent yields. The transformations tolerated a wide range of *N*-sulfonyl-1-aza-1,3-dienes with different C4-substituents, both aryl and alkyl. The addition of a Brønsted acid to the reaction system slightly improved the yields and diastereocontrol. In addition, the resulting tetrahydropyridines could be transformed to more complex dihydroxylated piperidine derivatives.

**Scheme 52:**
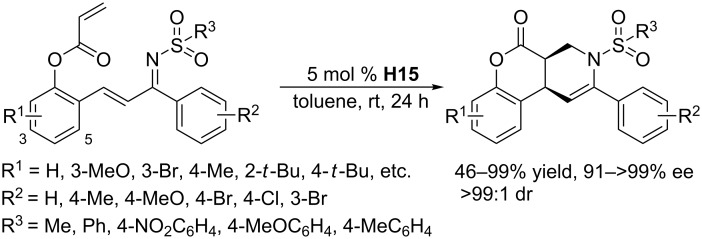
Intramolecular tandem RC/Michael addition, catalyzed by the chiral phosphine **H15**.

At almost the same time, an intramolecular variant of this [4 + 2] annulation was developed, employing the chiral bifunctional phosphine **H15** as the catalyst (Scheme 52) [95]. The substrates were constructed by installing an acrylate moiety and an α,β-unsaturated imine moiety on the aryl scaffold. In the presence of 5 mol % of **H15**, the functionalized substrates underwent the intramolecular [4 + 2] annulation in toluene at room temperature for 24 h to provide highly functionalized tetrahydropyridines in moderate to excellent yields with exceptionally high diastereo- and enantioselectivities. The optically pure products, containing multiple functional groups, could undergo further transformations, such as Diels–Alder reactions, reduction, and hydrolysis, to afford nitrogen-containing heterocyclic compounds. In contrast to the intermolecular aza-RC reaction/Michael addition sequence described above, the mechanism was assumed to involve an initial aza-RC reaction between the α,β-unsaturated imine and the enolate, followed by an S_N_2 reaction.

#### 2.14 Annulations through double-Michael additions

**Scheme 53:**
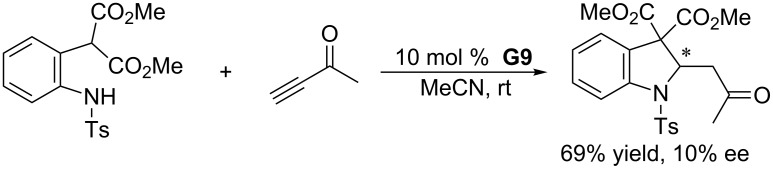
Double-Michael addition, catalyzed by the chiral aminophosphine **G9**.

The bisphosphine-catalyzed double-Michael addition of dinucleophiles to electron-deficient alkynes provides an efficient approach for the synthesis of biologically significant nitrogen-containing heterocycles. To develop its asymmetric variant, Kwon and co-workers examined several common and commercially available chiral bisphosphines, as well as a series of newly prepared chiral aminophosphines [96]. Unfortunately, these phosphines displayed no or very poor enantioselectivity. In a relatively successful example, the chiral aminophosphine **G9** catalyzed the asymmetric double-Michael reaction between *o*-tosylamidophenyl malonate and 3-butyn-2-one to give the indoline derivative in 69% yield and up to 10% ee (Scheme 53).

#### 2.15 Annulation through tandem Michael addition/Wittig olefination

**Scheme 54:**
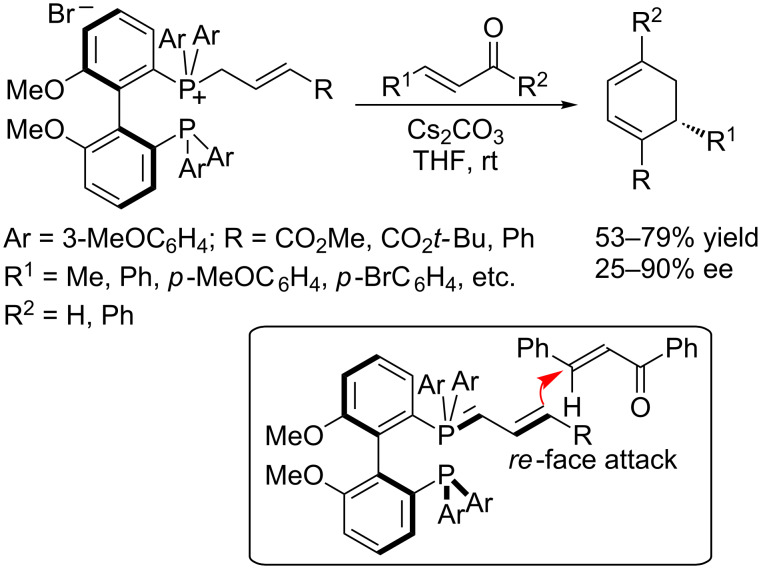
Tandem Michael addition/Wittig olefinations, mediated by the chiral phosphine BIPHEP.

In 2009, Tang and Zhou developed an annulation through tandem Michael addition/Wittig olefination, mediated by the chiral phosphine BIPHEP, for the synthesis of optically active cyclohexa-1,3-diene derivatives (Scheme 54) [97]. Although this reaction required a stoichiometric amount of chiral phosphine, it is quite interesting and deserves mention. In the presence of cesium carbonate, chiral BIPHEP-derived phosphonium ylides reacted with various α,β-unsaturated aryl/alkylketones in THF at room temperature to afford corresponding cyclohexadienes in good yields and with up to 90% ee. It was proposed that the major enantiomer formed through *re*-face attack of the ylide onto the Michael acceptor, rather than attack from the sterically hindered *si*-face.

#### 2.16 Michael additions

**Scheme 55:**
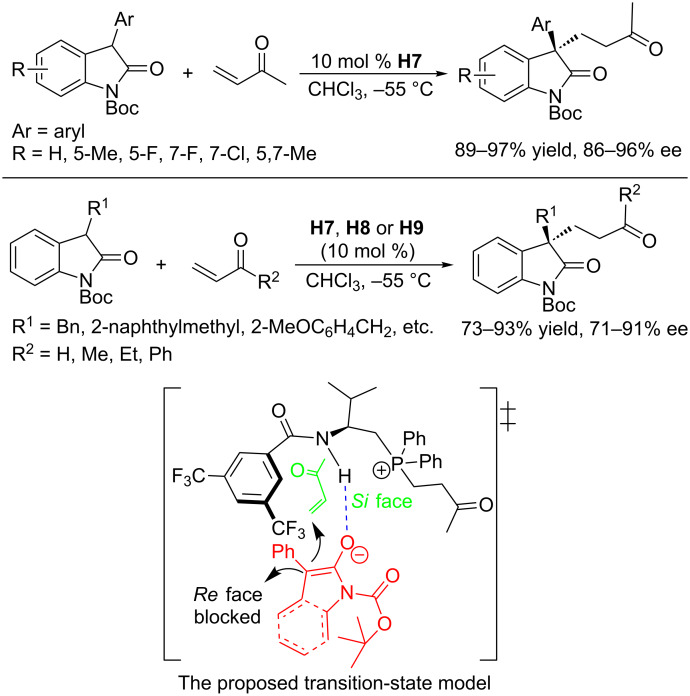
Asymmetric Michael additions, catalyzed by the chiral phosphines **H7**, **H8**, and **H9**.

Asymmetric Michael addition is one of the most studied enantioselective processes in organic synthesis, with many successful examples having been reported. Michael reactions employing nucleophilic phosphines are believed, however, to proceed through a mechanism involving phosphine-initiated general base catalysis; consequently, they do not involve covalent linkage of the phosphine to the reactants [98]. Accordingly, the development of chiral phosphine–assisted asymmetric Michael reactions has lagged behind other phosphine-catalyzed reactions. Recently, using bifunctional chiral amino acid–derived phosphines, Lu and co-workers developed asymmetric Michael additions of oxindoles to α,β-unsaturated carbonyl compounds (Scheme 55) [99]. They identified the chiral phosphine **H7** as the best catalyst, providing the corresponding Michael adducts in excellent yields and enantioselectivities. Notably, the less studied and relatively inert 3-alkyl-substituted oxindoles were also applicable to the reaction in the presence various chiral phosphines. When exposed to appropriate catalysts (**H7**, **H8**, or **H9**), 3-alkyl-substituted oxindoles reacted smoothly with Michael acceptors for an extended period of time, giving an array of corresponding products in good to excellent yields and enantioselectivities. In the proposed transition state, a hydrogen bond between the amide NH proton and the enolate oxygen atom assisted the Michael addition from the *si*-face of the enolate. The alternative *re*-face attack was blocked by the 3,5-bistrifluoromethylphenyl group (Scheme 55).

#### 2.17 γ-Umpolung additions of allenes or alkynes

In the phosphine-catalyzed annulations of allenoates and activated alkenes, the first C–C bond forms through nucleophilic addition of the β-phosphonium dienolate intermediate to the activated alkene at its α- or γ-carbon atoms. Conversely, when allenoates are mixed with pronucleophiles that contain acidic protons in the presence of a phosphine, the β-phosphonium dienolate zwitterion becomes protonated at its α-carbon atom, resulting in the formation of vinylphosphonium species and anionic nucleophiles. The nucleophile anion then undergoes γ-umpolung addition to the γ-carbon atom of the vinylphosphonium species, producing a phosphonium ylide intermediate. Subsequent proton transfer and β-elimination of the phosphine catalyst results in a γ-functionalized α,β-unsaturated enoate. The reaction, known as γ-umpolung addition, was reported by Trost [100] and Lu [101] in 1994 and 1995, respectively. Trost employed butynoates, which are converted to β-phosphonium dienolates under the conditions of phosphine catalysis.

**Scheme 56:**
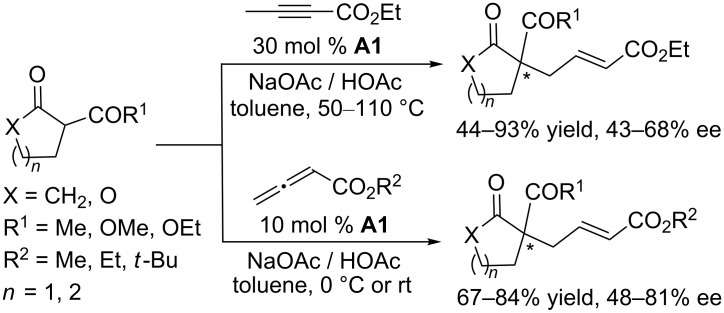
Asymmetric γ-umpolung additions, catalyzed by the chiral phosphine **A1**.

**Scheme 57:**
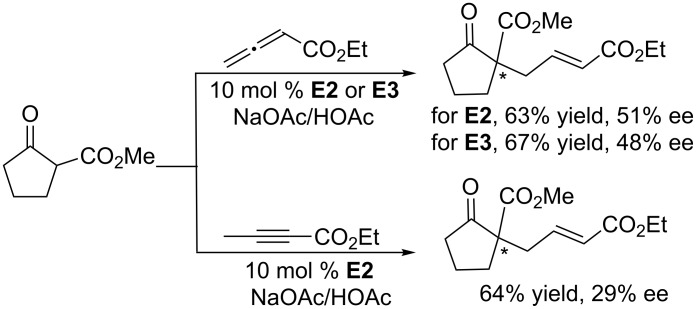
Asymmetric γ-umpolung additions, catalyzed by the chiral phosphines **E2** and **E3**.

Early in 1998, the Zhang group explored phosphine-catalyzed asymmetric γ-umpolung addition of 2-butynoates and allenoates (Scheme 56) [102]. Using the cyclic chiral phosphine **A1**, featuring a bridged-ring skeleton, as the catalyst and NaOAc/HOAc as additives, asymmetric γ-addition of cyclic β-dicarbonyl nucleophiles to 2-butynoate occurred in toluene at relatively high temperature to produce γ-adducts with quaternary carbon centers in good to excellent yields (up to 93%) and with moderate ee (up to 68%). Under the same conditions, the allenoates also underwent the γ-addition to give corresponding products in up to 84% yield and with up to 81% ee. In 2004, using the cyclic chiral phosphines **E2** and **E3**, based on a five-membered phospholane ring skeleton, as catalysts, Pietrusiewicz and co-workers reinvestigated these reactions (Scheme 57) [103]. The catalytic activities and enantiocontrol provided by these chiral phosphanes were, however, unsatisfactory, leading to only low to moderate ee.

**Scheme 58:**
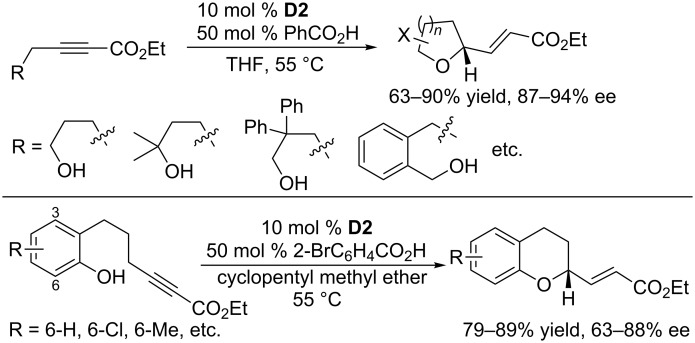
Intramolecular γ-additions of hydroxy-2-alkynoates, catalyzed by the chiral phosphine **D2**.

Although intermolecular γ-additions of 2-butynoates were quite unsuccessful, Fu and co-workers achieved successful γ-addition-based intramolecular annulations when using cyclic chiral phosphines featuring a spirocyclic skeleton (Scheme 58) [104]. In the presence of 10 mol % of **D2** and 50 mol % of benzoic/4-bromobenzoic acid as additives, γ-additions of a series of hydroxy-2-alkynoates occurred smoothly in THF at 50–55 °C to give substituted tetrahydrofurans, tetrahydropyrans, and dihydrobenzopyrans in good to excellent yields (63–90%) and enantioselectivities (87–94% ee). Both alkanol and phenol derivatives were compatible with this catalytic system.

**Scheme 59:**
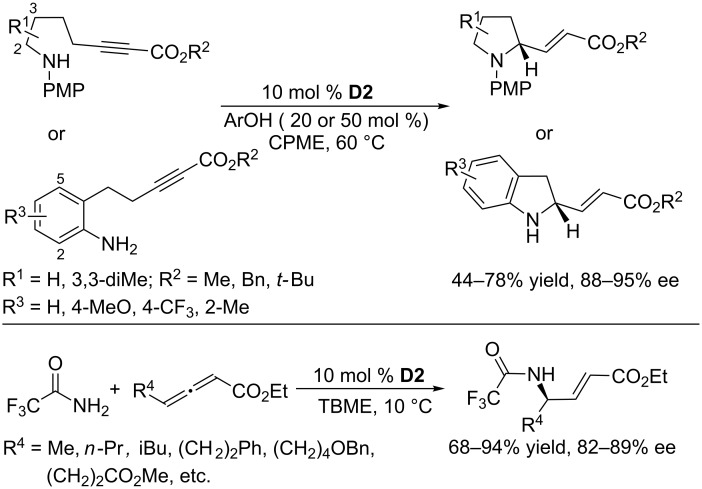
Intra-/intermolecular γ-additions, catalyzed by the chiral phosphine **D2**.

Building on the successful oxa-umpolung additions, Fu and co-workers further extended the reaction to intramolecular γ-additions of amino 2-alkynoates (Scheme 59) [105]. For this γ-addition of nitrogen nucleophiles, the chiral phosphine **D2** remained the most effective catalyst. In the presence of 10 mol % of **D2** and 2,4-dimethoxyphenol as an additive, intramolecular γ-additions of aromatic nitrogen nucleophiles to alkynoates occurred in cyclopentyl methyl ether at 60 °C to give functionalized pyrrolidines in moderate to good yields with excellent enantioselectivities (88–95% ee). A wide range of substrates was well-tolerated. Gratifyingly, intermolecular γ-addition of a nitrogen nucleophile to allenes was also possible; in the presence of 10 mol % of **D2**, the γ-addition reactions of 2,2,2-trifluoroacetamide to a variety of allenoates proceeded smoothly in *tert*-butyl methyl ether at 10 °C, leading to a wide range of α,β-unsaturated γ-amido carbonyl compounds in good to excellent yields and with high ee.

**Scheme 60:**
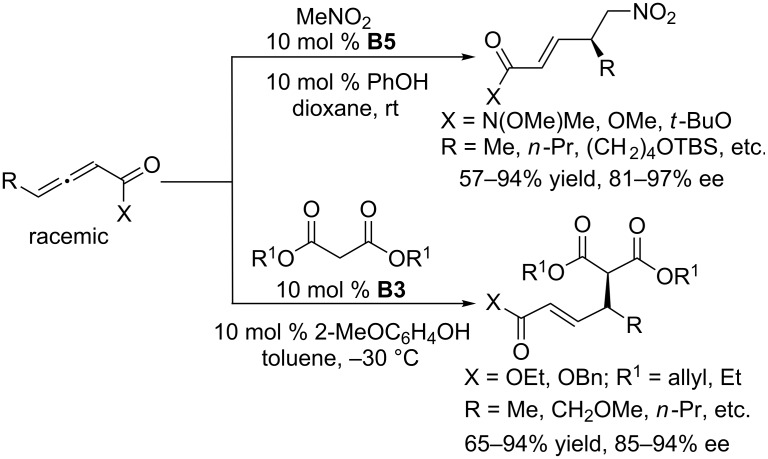
Intermolecular γ-additions, catalyzed by the chiral phosphines **B5** and **B3**.

Using cyclic chiral phosphines based on a binaphthyl skeleton, Fu and co-workers also achieved γ-additions of nitromethane to allenoates [106]. In the presence of 10 mol % of **B5** and the assistance of 10 mol % phenol, nitromethane underwent γ-additions to various allenoates in dioxane at room temperature (Scheme 60) to give corresponding α,β-unsaturated δ-nitro carbonyl compounds with good catalytic efficiency (57–94% yields, 81–97% ee). In the further exploration, using 10 mol % of the chiral phosphine **B3** as the catalyst and 10 mol % of 2-methoxyphenol as an additive, the γ-additions of malonate esters to allenoates were also successfully developed [107]. The γ-additions between a wide array of racemic allenoates and malonate esters proceeded well in toluene at –30 °C, furnishing a variety of the corresponding γ-substituted α,β-unsaturated esters in good yields with good to excellent enantioselectivities (Scheme 60). Notably, the α-substituted malonate esters could react with the allenoates to provide optically active α,β-unsaturated carbonyl compounds featuring two adjacent carbon stereocenters: a chiral quaternary carbon atom and a chiral tertiary carbon atom.

**Scheme 61:**
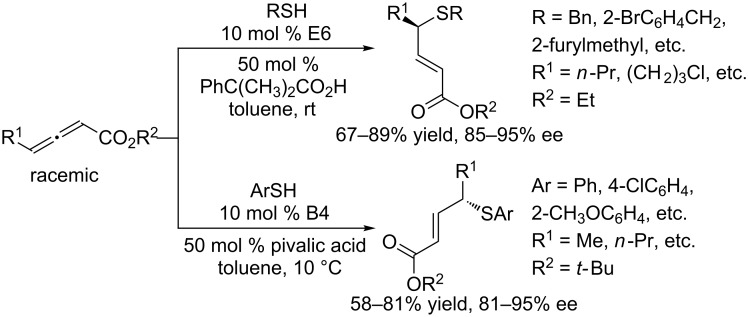
Intermolecular γ-additions, catalyzed by the chiral phosphines **E6** and **B4**.

In subsequent investigations, Fu and co-workers further demonstrated that alkyl and aryl thiols could also act as nucleophiles for asymmetric γ-additions to various allenoates [108–109]. In the presence of 10 mol % of TangPhos **E6** as the catalyst and 50 mol % of 2-methyl-2-phenylpropionic acid as an additive, alkyl thiols underwent γ-additions to various allenoates in toluene at room temperature to give γ-thioesters in 67–89% yields and with 85–95% ee (Scheme 61) [108]. Notably, the enantioselectivities were significantly additive-dependent. For the asymmetric γ-additions of aryl thiols to allenoates, the chiral binaphthyl phosphine **B4** proved to be the best catalyst [109]. In the presence of 10 mol % of **B4** as the catalyst and pivalic acid as an additive, the γ-additions between various aryl thiols and an array of allenoates progressed well in toluene at 10 °C to afford γ-arylthio-α,β-unsaturated esters in 58–81% yields and with 81–95% ee. This reaction provides facile access to various chiral alkyl aryl thioethers under mild conditions.

#### 2.18 Allylic substitutions of MBH acetates or carbonates with nucleophiles

In addition to various annulations, allylic substitution is another important class of reaction of MBH acetates or carbonates in nucleophilic phosphine catalysis that can be used to synthesize valuable molecules.

**Scheme 62:**
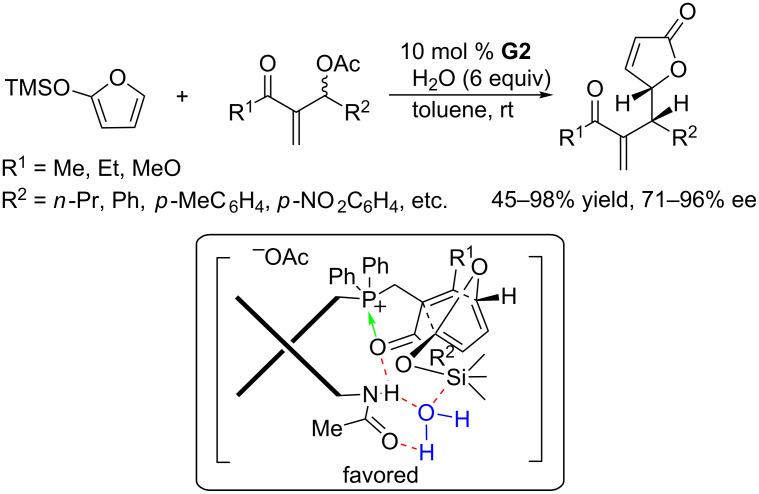
Asymmetric allylic substitution of MBH acetates, catalyzed by the chiral phosphine **G2**.

For the synthesis of γ-butenolide ring systems, which are very common structural motifs in naturally occurring organic molecules, the Krische group developed PPh_3_-catalyzed allylic substitutions of MBH acetates with 2-(trimethylsilyloxy)furan [110–111]. Using the bifunctional chiral phosphine **G2**, Shi and co-workers accomplished the asymmetric variant of this reaction (Scheme 62) [112]. In the presence of 10 mol % of **G2** and excess water (6 equiv), which was assumed to function as an extra proton source, the allylic substitutions of MBH acetates bearing either aromatic or aliphatic substituents at the allylic position with 2-(trimethylsilyloxy)furan proceeded smoothly in toluene to afford enantioenriched γ-butenolides in moderate to excellent yields and with excellent enantioselectivities. An intermediate arising from *endo*-selective Diels–Alder annulation of the siloxy-furan complex with the enone was believed to play a key role in the enantiocontrol (Scheme 62). In the intermediate, a molecule of water served as a bridge of hydrogen bonds to connect the amide NH proton of the phosphonium ylide to the silicon atom of the trimethylsilyloxy group, which directed the approaching furan moiety to the Michael acceptor in an *endo* manner.

**Scheme 63:**
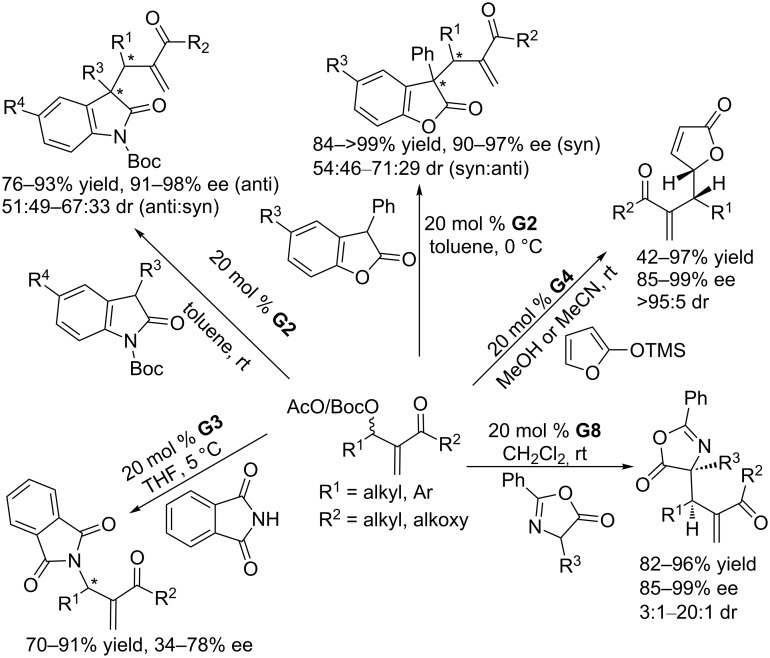
Allylic substitutions between MBH acetates or carbonates and an array of nucleophiles, catalyzed by chiral binaphthyl-derived multifunctional phosphines.

Using multifunctional chiral phosphines based on a binaphthyl skeleton, Shi and co-workers explored the phosphine-catalyzed asymmetric allylic substitutions of MBH acetates or carbonates with various nucleophiles, both experimentally and theoretically. In the presence of chiral phosphines presenting tethered amide or thiourea moieties (**G2**, **G3**, **G4**, **G8**), various activated nucleophiles, including phthalimide [113–114], oxazolones [115], and benzofuran-2(3*H*)-ones and oxindoles [116], could undergo substitution reactions with MBH acetates or carbonates, generating a variety of optically active MBH adducts in good yields and stereoselectivities (Scheme 63). Theoretical calculations were performed to explore the origins of stereoselectivities and to confirm Shi’s previously proposed mechanism [117]. The MP2/6-31G(d)//HF/3-21G* level of theory was used to calculate and compare the energies of the transition states. The calculations revealed that the energy of the transition state for *endo*-Diels–Alder [4 + 2] annulation was the lowest among the four possible transition states, presumably arising from the π–π-stacking interactions; these energy gaps likely account for the diastereoselection. Furthermore, the enantioselectivity was ascribed to the energy difference between the transition states for the two possible faces of attack (*re*- and *si*-endo transition states), on the basis of optimized structures. The *si*-endo transition state was disfavored because of additional repulsion between the trimethylsilyloxy unit and the phosphine, as well as the absence of hydrogen bonding between the amide NH proton and the C=O group in the MBH adduct.

#### 2.19 Asymmetric acylations of alcohols

**Scheme 64:**
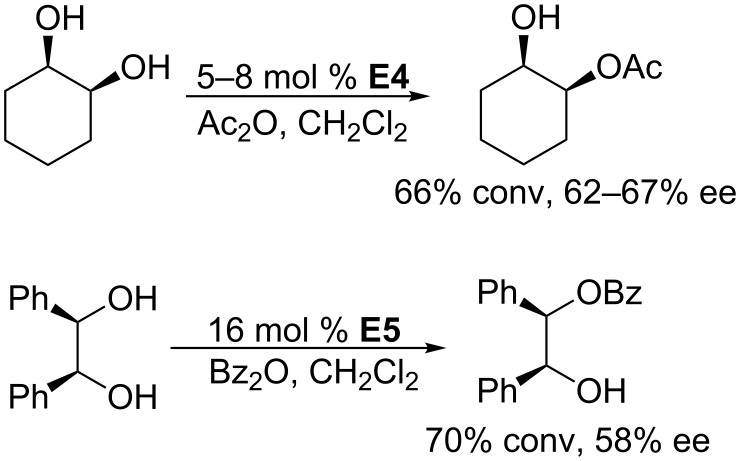
Asymmetric acylation of diols, catalyzed by the chiral phosphines **E4** and **E5**.

Kinetic resolution of a racemic mixture is a powerful tool for the synthesis of enantioenriched compounds. Among the various methods, including enzyme catalysis, metal catalysis, and organocatalysis, that have been developed for this process, phosphine catalysis is particularly interesting. In 1996, Vedejs and co-workers reported the first example of chiral phosphine-catalyzed enantioselective acylation based on kinetic resolution (Scheme 64) [19]. They demonstrated that the cyclic chiral phosphines **E4** and **E5** catalyze the acetylation and benzoylation of secondary alcohols effectively in dichloromethane, yielding corresponding esters in moderate to good conversions and with moderate enantioselectivities. For instance, in the presence of 5–8 mol % of **E4**, the desymmetrization reaction of *cis*-1,2-cyclohexanediol with acetic anhydride in dichloromethane at 0–20 °C gave the monoacetate in 66% conversion and with 62–67% ee. Using 16 mol % of **E5**, the reaction of *meso*-hydrobenzoin with benzoic anhydride afforded the monobenzoate in 70% conversion and with 58% ee.

**Scheme 65:**
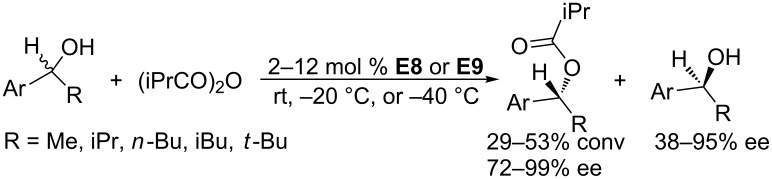
Kinetic resolution of secondary alcohols, catalyzed by the chiral phosphine **E8** and **E9**.

In a subsequent study, Vedejs and co-workers developed more efficient and enantioselective chiral phosphines – **E8** and **E9** – for the kinetic resolution (Scheme 65) [20]. In the presence of 2–12 mol % of **E8** or **E9**, racemic secondary alcohols reacted with (iPrCO)_2_O in heptane to provide isobutyrates in moderate conversions and with good to excellent enantioselectivities. At the same time, the starting materials were kinetically resolved into corresponding enantioenriched alcohols. Moreover, this kinetic resolution could be performed on the gram-scale.

## Conclusion

This review reveals that, in less than two decades, tremendous progress has been made in the study of asymmetric reactions catalyzed by nucleophilic chiral phosphines. These reactions have emerged as attractive and powerful synthetic tools, allowing the convenient preparation of many enantioenriched and functionalized carbocycles, heterocycles, and acyclic compounds in satisfactory yields. Nevertheless, the range of successful asymmetric reaction types remains relatively limited. Although many chiral phosphines have been prepared, the number of appropriate and available chiral phosphines is small when compared with those that mediate achiral reactions. Because of the lack of suitable chiral catalysts, asymmetric variants of many phosphine-catalyzed reactions remain unsubstantiated. Even for those successful asymmetric reactions, quite a few catalyst systems are substrate-dependent and do not work for slightly different, yet analogous, substrates. Based on these issues, the design and synthesis of novel chiral phosphines, including cyclic phosphines and multifunctional chiral phosphines, remains an interesting challenge. Because of the powerful capabilities of phosphine-catalyzed reactions in the synthesis of biologically active molecules and natural products, there is a need for further research in this area, such that more asymmetric reactions can be anticipated.
